# Chemical Feedback and Collective pH-Dynamics in Enzyme-Powered Artificial Cells

**DOI:** 10.1021/acs.jpcb.6c03926

**Published:** 2026-09-21

**Authors:** Nils Göth, Joachim Dzubiella

**Affiliations:** † Applied Theoretical Physics-Computational Physics, Physikalisches Institut, Albert-Ludwigs-Universität Freiburg, D-79104 Freiburg, Germany; ‡ Cluster of Excellence livMatS @ FIT - Freiburg Center for Interactive Materials and Bioinspired Technologies, Albert-Ludwigs-Universität Freiburg, D-79110 Freiburg, Germany

## Abstract

Chemical communication between artificial cells (ACs) is crucial for the collective behavior in synthetic prototissues. Recently synthesized ACs show pH-responsive and pH-modifying behavior by an enzymatic chemical reaction. However, the chemical interplay between pH-responsive ACs is theoretically not well studied. Here, we report a simple two-variable model for such ACs that couples substrate conversion, enzyme activity, membrane permeability, and pH-buffering. We calibrate the model to recent experiments of glucose oxidase- and urease-loaded ACs, and reproduce a protection mechanism against external acid or base perturbations. Building on the derived models we predict the dynamics of AC-mixtures of the two antagonistic ACs. Depending on composition and initial pH, the mixtures exhibit a stable low-pH state, a stable high-pH state, or bistability, providing a minimal route to hysteretic pH control in AC-communities. On longer time scales, fuel depletion leads to transient states with programmable pH and lifetime. Our results demonstrate the potential of chemical communication in AC-systems and provide testable design rules for future experiments.

## Graphical abstract

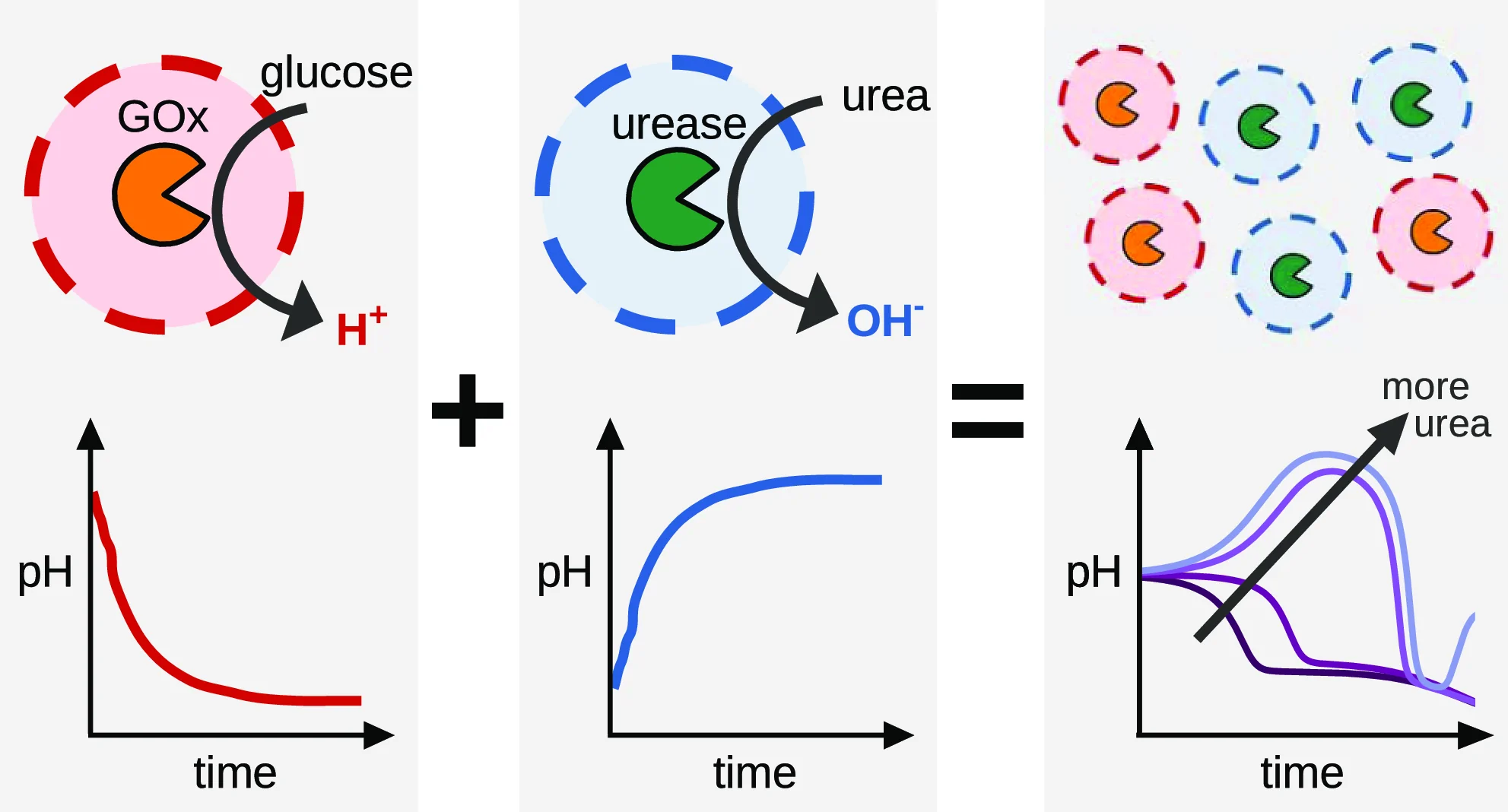

## Introduction

In living matter, chemical communication between cells and bacteria is vital. Bacteria release small, freely diffusing molecules and measure their density to estimate the local bacterial density; this is called quorum sensing.^1^ They use quorum sensing to coordinate collective behavior, for example in biofilm formation^2^ and the distribution of public goods.^3^ Inspired by nature, modern material science aims at engineering chemical communication, in particular between artificial cells (ACs).^4–7^ The communication enables complex dynamics such as collective behavior,^8^ distribution of tasks,^9^ and enzyme cascade reactions.^10^ Even more complex biomimetics are possible when there are more than one type of AC communicating with each other.^10–13^

Enzyme-powered ACs are one possible way to enable chemical communication between ACs.^14,15^ The enzyme is trapped inside the AC and converts a fuel into a signal which can diffuse through the AC membrane and reach the other ACs. An important group of enzymes comprises those that catalyze acid- or base-producing reactions due to their effect on pH. Examples include glucose oxidase, which catalyzes the reaction from glucose to gluconic acid,^16–18^ and urease, which catalyzes the reaction from urea to ammonia.^19–21^

ACs can be liposomes,^22^ organelles,^23,24^ proteinosomes,^25^ or polymer-based.^7^ The advantage of polymer-based ACs is their longevity and stability.^7^ The polymer network forming the membrane can be pH-responsive and thus enables a swelling-collapse transition with pH-dependent membrane permeability.^11,26,27^ Thus, an AC does not only respond to its own pH-signal but also to the signal of the neighboring ACs—which is chemical communication. This can lead to different collective effects, e.g., behavior similar to the quorum sensing of bacteria.^8^

In a recent study, Krehan et al. synthesized ACs to control the pH of the bulk solution,^11^ as sketched in Figure 1a. The ACs have a pH-responsive polymer membrane based on poly­(methacrylic acid-*co*-N-butylmethacrylamide) (PMAA) with a diameter of about 70 μm. Inside the ACs an enzyme, glucose oxidase (GOx), is trapped due to its size. The aqueous system is initialized at high pH and fueled with glucose. Initially, the glucose diffuses through the membranes into the ACs and reacts enzymatically with GOx to gluconic acid. The produced gluconic acid diffuses back out of the ACs and thereby lowers the pH of the bulk solution. When the pH reaches a lower critical value, the pH-responsive membranes collapse, making the ACs impermeable to glucose. Thus, no more gluconic acid is produced and the pH decrease stops (see subplot in Figure 1a). This differs from the system where the GOx is free in the solution and thereby enables much lower pH. In addition, Krehan et al. synthesized inverse working ACs based on the urease–urea reaction and a poly­(2-(diethylamino)­ethyl methacrylate)-membrane, sketched in Figure 1b. These urease-ACs raise the pH until the membranes collapse at a high pH and thereby stop the reaction.

**Figure 1:**
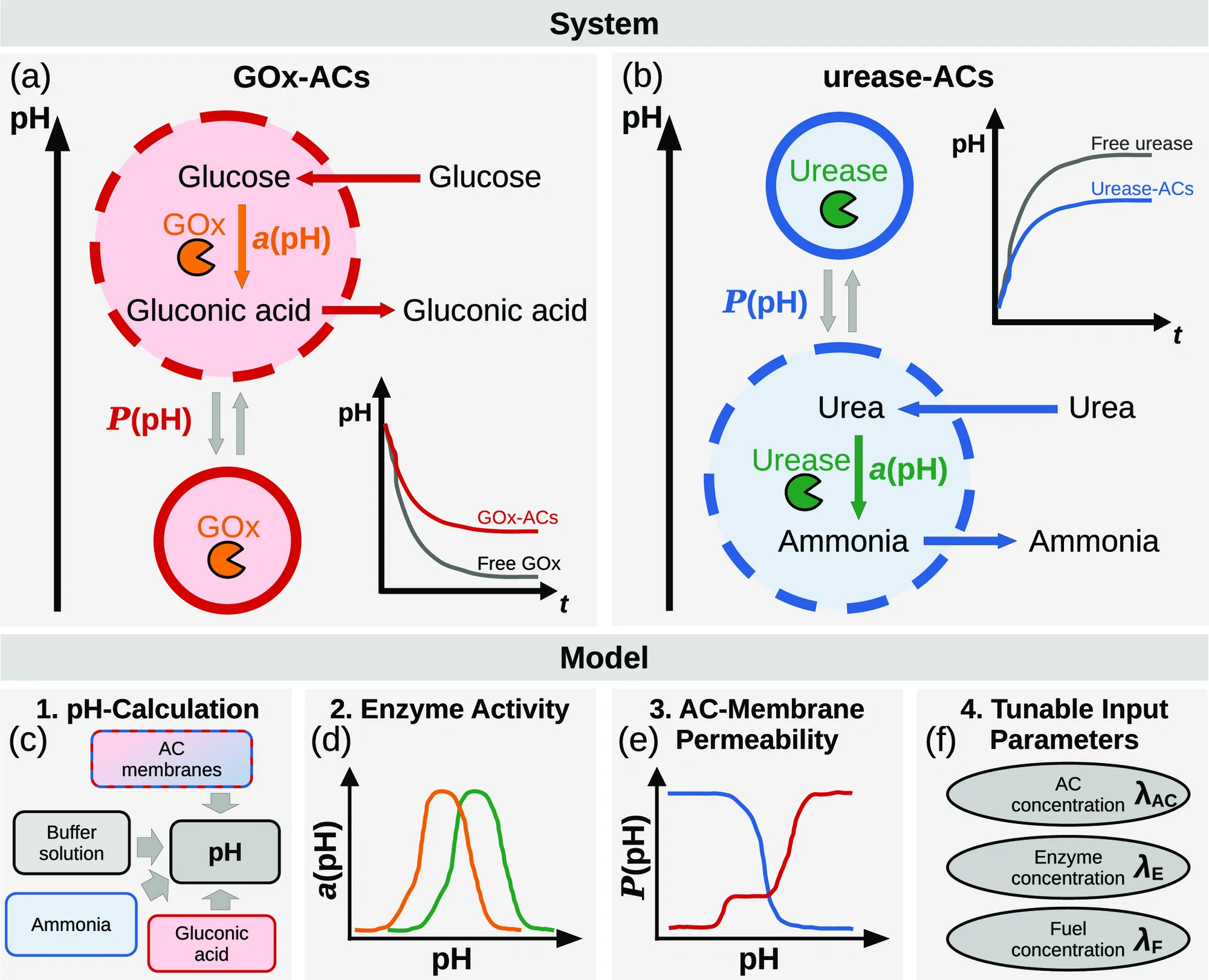
Sketch of the system and the model. (a) GOx-ACs are swollen/permeable at high pH and collapsed/impermeable at low pH. In the permeable state glucose can enter the ACs, react with GOx to gluconic acid, and the gluconic acid can diffuse out. The inset graph sketches the pH evolution for GOx inside the ACs (red) and in the free solution (gray). (b) Urease-ACs are collapsed/impermeable at high pH and swollen/permeable at low pH. In the permeable state urea can enter the ACs, react with urease to ammonia, and the ammonia can diffuse out. The inset sketches the pH evolution for urease inside the ACs (blue) and in the free solution (gray). The model consists of (c) the pH calculation depending on the different ingredients, (d) the enzyme activity *a*(pH) of GOx (orange) or urease (green), (e) the membrane permeability term *P*(pH) of the GOx-ACs (red) or urease-ACs (blue), and (f) the effects of tunable input parameters.

Alongside experiments, theoretical models of enzyme-powered ACs are needed to efficiently study the high-dimensional parameter spaces of such systems.^4^ Existing models of enzyme-powered ACs focus on the theoretically possible autonomous pH oscillations. These oscillations are a result of the bell-shaped pH-activity curves of the enzyme in the urea–urease system^28–30^ and the ester–esterase system.^31^ Another theoretical possibility for autonomous oscillations is a pH-dependent membrane permeability,^32,33^ with existing experimental indications in ACs with an internal glucose–glucose oxidase reaction.^26^ Furthermore, chemical communication between enzyme-powered ACs can yield synchronization of pH-clocks^34^ or collective oscillations.^35^ However, models which explicitly include changes in the bulk solution focus on lipid vesicles.^15,34^ Consequently, the collective feedback between the bulk solution and the polymer-membrane permeability remains unexplored. Additionally, to the authors’ knowledge there are no existing models describing the interplay between antagonistic ACs. Itatani et al. showed in experiments that even the interplay between an acid- and a base-producing enzyme (without ACs) leads to rich behavior due to the antagonistic products.^19^ This motivates a more detailed analysis of enzyme-powered AC-mixtures with pH-dependent membrane permeabilities to complement recent experiments.^11,12^

Here, we propose a model to study the collective behavior of (i) acid or base producing ACs and (ii) their mixtures. The model is a simple two-variable model of the substrate and product concentration in the solution. The two concentrations are coupled via a reaction rate which includes the pH-dependent enzyme activity and the pH-dependent membrane permeability. We calibrate our model to the recent experiments by Krehan et al.^11^ and fit the free parameters to the experimental data. In the following, we will present the model on the basis of GOx-ACs and show that it also works for urease-ACs. Thus, the model enables predictions for a high dimensional parameters space and thereby enable less experimental search in the future. In addition, the model is also capable to qualitatively cover the protection mechanism, in which the ACs stabilize the pH value against external perturbations. Finally, we apply our two fitted models to AC-mixtures of GOx-ACs and urease-ACs. We find bistability in pH, transient states, and dynamics on different time scales. Thus, our work demonstrates the rich behavior of AC-mixtures and can be used as a guide for future experiments.

## Model and Methods

Our model aims to describe the time-resolved pH evolution of enzyme-powered AC-systems. We calibrate the model to experiments with GOx-ACs performed by Krehan et al.^11^ The function of the GOx-ACs is sketched in Figure 1a. The model for the urease-ACs (Figure 1b) is based on the same experiments^11^ and is obtained analogously in the Supporting Sections S1–S3 with Figures S1–S4.

The main chemical reaction in the GOx-ACs is the enzymatic catalysis by GOx of

glucose(glu)⁡⁡→⁡⁡gluconicacid(ga).

In fact, the enzyme GOx catalyzes the reaction from oxygen and glucose to glucono-δ-lactone (GDL) which in turn spontaneously hydrolyzes into gluconic acid.^36,37^ The slowest time scale in this reaction chain is the hydrolysis of GDL (≲1 h).^36^ Since this is still significantly faster than the pH change observed in the experiments with ACs (∼3 h), the transient GDL step is skipped for simplicity. We thereby also ignore the inverse reaction from gluconic acid to GDL, which is justified by the low ratio of GDL to gluconic acid in the steady state (<10%) in the studied pH range.^38^ Additionally, we neglect diffusional effects in the bulk and assume homogeneously distributed chemical concentrations, because the experimental container has dimensions of millimeters and is shaken regularly. This also applies to the oxygen concentration, which we assume is always saturated; therefore, an oxygen saturation term, as used in ref 16 is omitted. We further merge the three steps of glucose diffusing into the ACs, glucose reacting to gluconic acid, and gluconic acid diffusing out of the ACs, into one single step. The reason is that the experimental data show no clear indications of a membrane response time (e.g., no oscillations). Thus, we assume that the pH within the ACs and outside the ACs (bulk solution) is identical. This assumption is supported by experimental measurements in the urease-AC system, which show similar values (see Figure 3P in ref 11). This results in two rate equations for the bulk glucose concentration *c*_glu_ and gluconic acid concentration *c*_ga_ (in mM)

1a $$\frac{dc_{glu}}{dt}=-\frac{dc_{ga}}{dt},$$

1b $$\begin{array}{ll} \frac{dc_{ga}}{dt} & =k_{GOx}a\left(pH\right)P\left(pH\right)B_{ref}\\ & \frac{N_{G‐AC}}{\lambda_{G‐AC}+N_{G‐AC}}\frac{\rho_{GOx}}{\lambda_{GOx}+\rho_{GOx}}\frac{c_{glu}}{\lambda_{glu}+c_{glu}}. \end{array}$$

Equation 1a describes the stoichiometry of the relevant reactant and product. Equation 1b consists of the reaction rate *k*_GOx_, which is reduced by the pH-dependent activity of the enzyme GOx *a*(pH), the permeability of the pH-responsive membranes *P*(pH), and the finite concentrations of ACs *N*_G‑AC_, GOx in each AC ρ_GOx_, and glucose *c*_glu_. The latter three are modeled via a Langmuir ansatz with the half-saturation constants λ_*i*_. The reference constant $B_{ref}=\frac{\lambda_{G‐AC}+N_{G‐AC}^{r}}{N_{G‐AC}^{r}}\frac{\lambda_{GOx}+\rho_{GOx}^{r}}{\rho_{GOx}^{r}}\frac{\lambda_{glu}+c_{glu}^{r}}{c_{glu}^{r}}$ simplifies the calibration by eliminating the three Langmuir terms for a set of reference parameters and high glucose concentrations. The reference system in the experiments^11^ and in our model uses *N*_G‑AC_^r^ = 16/μL, the GOx concentration per AC $\rho_{GOx}^{r}=10g/L$ , and the initial glucose concentration $c_{glu}^{r}=100mM$ .

The differential equation system (1), is solved numerically with the Euler method and a time step of 0.005 h. We use the initial variables *c*_ga_(*t* = 0) ≡ *c*_ga,0_ = 0 and *c*_glu,0_, where the latter is varied. In the experiments^11^ they studied separately the effect of the AC concentration, the GOx concentration per AC, and the initial glucose concentration. In the following, we assemble the model details for this AC system in four steps as shown in Figures 1c–f: First, we calculate the pH value from the gluconic acid concentration and other chemicals in the solution. Second, we define the pH-dependent activity of the enzymatic reaction. Third, we determine the membrane permeability *P*. Fourth, we fit the terms containing the tunable input parameters *N*_G‑AC_, ρ_GOx_, and *c*_glu_. Parameters and technical details are in the Supporting Information Table S1 and Section S3.

## Results and Discussion

### Calibrating the Model

#### pH Calculation

The pH of the solution is affected by the reaction product (gluconic acid), the background buffer solution, and the AC-membranes. We obtain the pH via pH = −log_10_([H^+^]/M) with the proton concentration in the solution [H^+^]. Since the H_2_O concentration is high, we write [H^+^] instead of [H_3_O^+^]. To calculate the proton concentration, we numerically solve the charge balance equation which sums up all charges (protons, hydroxide ions, ionized acids and bases)^39^

2 $$\left[H^{+}\right]-\frac{K_{w}}{\left[H^{+}\right]}-\sum_{i}\frac{c_{acid,i}K_{a,i}}{K_{a,i}+\left[H^{+}\right]}+\sum_{j}\frac{c_{base,j}K_{b,j}}{K_{b,j}+K_{w}/\left[H^{+}\right]}=p_{0}$$

where *K*_w_ = 10^–14^ M^2^ is the self-ionization constant of water. The two sums run over all weak acids *i* and bases *j*, with the respective molar concentrations *c*_acid/base,*i*/*j*_ and acid/base dissociation constants *K*_a/b,*i*/*j*_. The constant *p*_0_ accounts for the charge of all strong acids and bases in the system which are therefore always fully ionized. The value of *p*_0_ is obtained by solving eq 2 for the known initial configuration, using [H^+^] according to the experimental initial pH.

In the experiments, the initial pH value of 7.35 is stabilized by adding a buffer solution to the system. The used 10 mM Dulbecco’s Phosphate Buffered Saline (DPBS) buffer solution is phosphate-based. It consists of an acid and a base with known concentrations^40^ and known acid/base dissociation constants. Thus, the buffer solution effect is incorporated into eq 2. In experiments with a lower initial pH, a strong acid (HCl) is also added to the solution, which is automatically accounted for in *p*_0_.

Polymer-based hydrogels buffer the pH.^41,42^ The PMAA in the AC membranes contains carboxyl groups, which lead to the AC collapse around pH 6 due to the protonation of the polymer’s carboxyl groups. Thereby, the membranes act as an additional buffer. For simplicity, we treat the AC-buffer behavior as a normal weak acid in eq 2, even though it has been shown that the exact behavior is more complex.^43^ We use p*K*_a,PMAA_ = 6.25 based on experiments with PMAA polymer brushes.^44^ For the acid concentration we approximate *c*_PMAA_ = 1.75·10^–10^ mol/AC by an estimate from geometry and membrane composition.^45^

With the charge balance eq 2, we approximate all ionization and neutralization processes of acids and bases as being at steady state. Other models using this approximation derive the effects from ionization rate constants.^28,29^ Instead, we use the acid dissociation constants which enable the integration of the membrane effects without knowing their ionization rate constants.

#### Enzyme Activity

The activity of enzymes depends strongly on pH (see Figure 1d). The activity curves of many enzymes exhibit bell-shaped behavior,^16,46^ including GOx.^16^ Motivated by the ionization constants of the substrate–enzyme complex, *K*_es1_ and *K*_es2_, we model this bell-shaped behavior by the commonly used expression^19,47^

3 $$a\left([H^{+}]\right)={\left(1+\frac{K_{es2}}{\left[H^{+}\right]}+\frac{\left[H^{+}\right]}{K_{es1}}\right)}^{-1}$$

The resulting profile has a standard bell-shape (Figure S1). We use the ionization constants of GOx obtained from experiments, *K*_es1,GOx_ = 7.94·10^–5^ M and *K*_es2,GOx_ = 3.98·10^–8^ M.^16^ The model yields a maximum GOx activity at pH 5.8, which is in agreement with other experiments.^17^ The manufacturer value for the maximum GOx activity cannot be used, as it is unknown to what extent the encapsulation process alters the activity.

#### Permeability Term

The pH-dependent permeability of the membrane for the relevant molecules cannot be derived from the membrane composition and geometry. Existing models treat variable membrane permeability via explicit hysteresis,^48^ a product-dependent reaction rate,^33,35^ or a permeability coefficient that varies with membrane potential.^15^ Instead, we take a single experimental curve, convert it into a pH-dependent permeability, and fit a smooth function *P*(pH) to it; see Figure 2. This permeability term bundles the permeability effects of glucose entering and gluconic acid leaving the AC into a single term *P*(pH). This yields a model for the specific experimental conditions and further enables us to gain insight into the membrane permeability.

**Figure 2:**
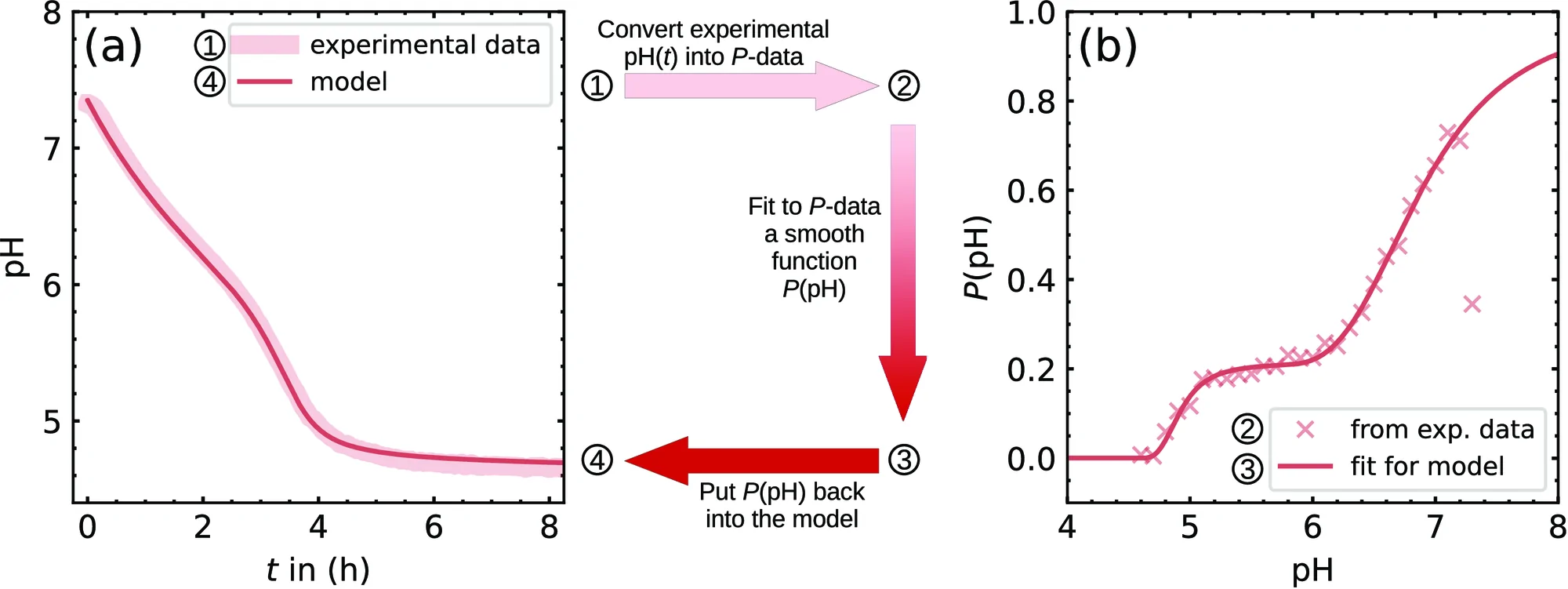
Determination of the permeability term of the GOx-AC membranes. (a) Time evolution of pH in the experiments^11^ (light red) and in the model (dark red). (b) Permeability term as a function of pH. The crosses are obtained from the experimental data using the model eq 1b. The solid line is a fit to this data. The data point at pH 7.3 is excluded from the fit because it involves the start of the experiment. The fitted *P*(pH) is put back into the model, which then results in the modeled pH evolution in (a). The experimental data in (a) is reproduced from ref 11 used under CC-BY 4.0 license. Copyright 2024 Joshua Krehan et al. (http://creativecommons.org/licenses/by/4.0/).

As experimental data we use the reference curve (*N*_G‑AC_ = 16/μL, ρ_GOx_ = 10 g/L, *c*_glu,0_ = 100 mM) shown in Figure 2a. From the experimental data we calculate with eq 2 the gluconic acid concentration *c*_ga_(*t*) according to the model. By taking the time derivative ${ċ}_{ga}$ we obtain the left-hand side of eq 1b. The three λ_*i*_-terms in eq 1b approximately eliminate *B*_ref_ for the high initial glucose concentration of *c*_glu,0_ = 100 mM. Therewith, we plot $P_{data}\left(pH\right)\propto {ċ}_{ga}\left(pH\right)/a\left(pH\right)$ up to the unknown prefactor *k*_GOx_ in Figure 2b. We observe a double-sigmoidal increase. We fix the impermeability limit *P*(4.6) = 0 and fit a double-sigmoidal function *P*(pH) to the data. Normalizing max­{*P*} = 1 yields the prefactor *k*_GOx_ = 0.00816/h. This *P*(pH) will be used as permeability term for all models with GOx-ACs. Finally, we put the obtained *P*(pH) into eq 1b of the model. Figure 2a shows that the fitted model matches the experimental data very well.

The double-sigmoidal behavior in Figure 2b reveals increases around pH 4.8 and 6.7. The latter coincides with the reported swelling-collapse transition of the GOx-ACs (Figure S5). Plotting *P* as a function of AC diameter (Figure S5e) shows a superexponential increase in this range, which agrees with theoretically predicted relations between permeability and polymer volume fraction.^49^ The increase in *P*(pH) around pH 4.8 occurs without a change in the AC diameter. We suspect conformational changes in the polymer network caused by the ionization of carboxyl groups, since the acid dissociation constant of MAA monomers is p*K*_a_ = 4.65.^50^

In principle, *a*(pH)*P*(pH) can be combined in a single term and a single fit. We decided to separate the two different effects to obtain information about the membrane permeability and check the reasonableness of the result.

#### Calibrating the Effects of Tunable Input Parameters

The other important parameters in the model are the AC concentration *N*_G‑AC_, the enzyme concentration ρ_GOx_, and the glucose concentration *c*_glu_(*t*). All three parameters or variables are proportional to the reaction rate for respective low concentrations. For high concentrations the reaction rate will saturate due to AC interactions or abundance of GOx or glucose. In a simple approach we neglect correlations between the parameters and use a Langmuir ansatz, *x*/(λ + *x*), for each term (see eq 1b). The free parameter λ is the concentration at which the reaction rate reaches half its maximum considering only the respective parameter. We fit all three Langmuir constants, λ_G‑AC_, λ_GOx_, λ_glu_, separately to experimental data by applying a least-squares minimization of the entire pH evolution (Figure 3). The term *B*_ref_ in eq 1b guarantees that the already obtained reference curve in Figure 2a does not change; cf. red line in Figures 3a–c. All three saturation terms are necessary to fit the experimental data.

**Figure 3:**
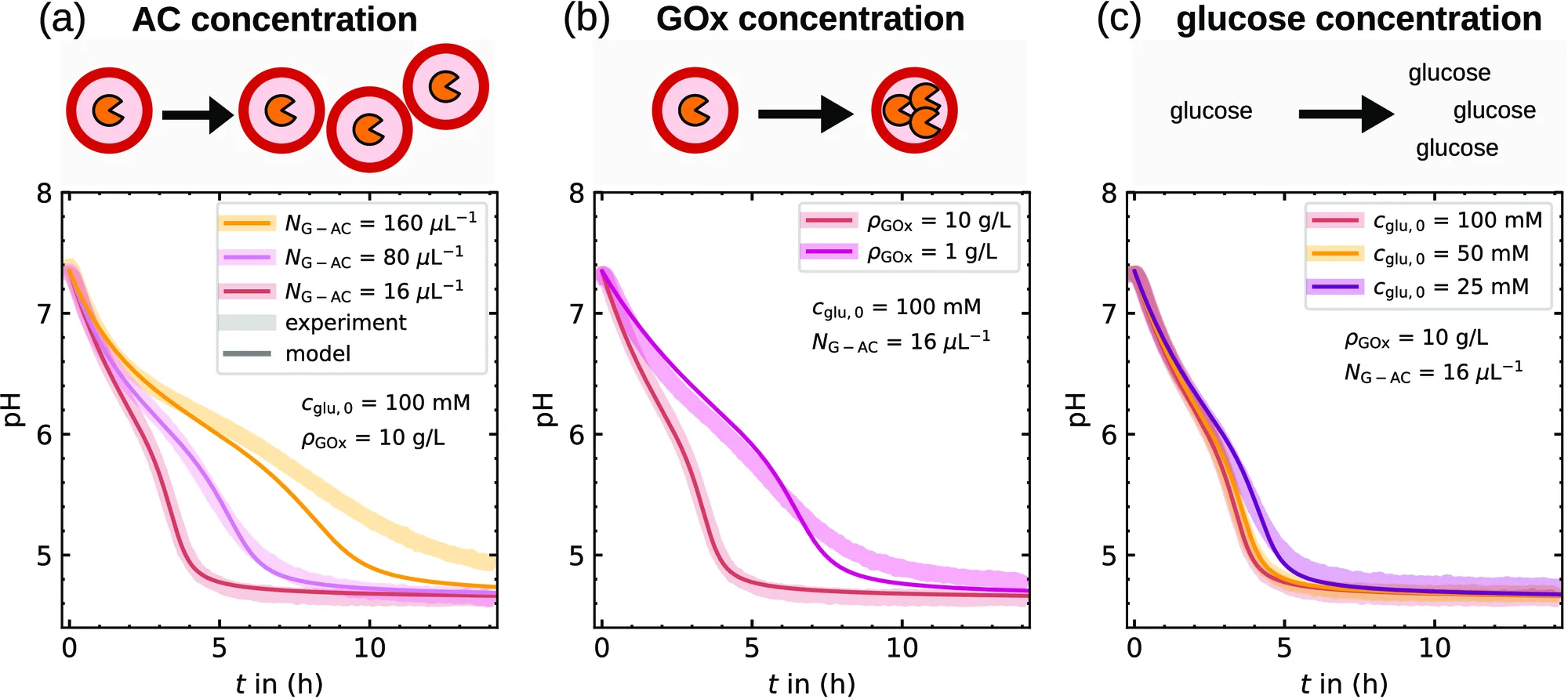
pH evolution for GOx-ACs and different input parameters. Variation of (a) AC concentration *N*_G‑AC_, (b) GOx concentration ρ_GOx_, and (c) initial glucose concentration *c*_glu,0_. The wide lines correspond to the experimental data,^11^ the thin lines to the model. The reference system used to fit *P*(pH) is always shown in red. The parameters λ_*i*_ are fitted to the data with (a) *N*_G‑AC_ = 80 μL^–1^, (b) ρ_GOx_ = 10 g/L, and (c) *c*_glu,0_ = 25 mM. The other remaining data in (a) and (c) serve as model validation. The experimental data is reproduced from ref 11 used under CC-BY 4.0 license. Copyright 2024 Joshua Krehan et al. (http://creativecommons.org/licenses/by/4.0/).

Figure 3a shows the pH evolution for different AC concentrations *N*_G‑AC_. The Langmuir constant is fitted to the experimental curve with 80 ACs/μL (pink) resulting in λ_G‑AC_ = (2.97 ± 0.05) μL^–1^. The fitted model matches well with its experimental data. The curve with 160 ACs/μL (orange) serves as a validation curve. We observe for the validation data set that the model shows a slightly faster pH decrease than the data, but in general matches the behavior. Interestingly, more ACs lead to a slower pH decrease. This can be explained by the buffer effect of the AC-membranes. The buffer capacity increases linearly with the AC concentration, while the reaction rate saturates around λ_G‑AC_ = 2.97 μL^–1^. An analysis reveals that the fastest pH decrease can be reached with 13 ACs/μL (Figure S6). Less ACs slow down the pH decrease due to the fixed 10 mM buffer solution.

Figure 3b shows the pH evolution for different enzyme concentrations ρ_GOx_. The Langmuir constant is fitted to the experimental curve with 1 g/L GOx (pink) resulting in λ_GOx_ = (1.141 ± 0.018) g/L and matching the experimental data well. Since no experimental data exists for a third enzyme concentration, there is no validation curve as for the AC concentration. In general, higher GOx concentrations lead to faster pH decreases which is expected.

Figure 3c shows the pH evolution for different initial glucose concentrations *c*_glu,0_. The Langmuir constant is obtained from a fit to the experimental curve with 25 mM glucose (purple) resulting in λ_glu_ = (5.88 ± 0.15) mM. It matches the data very well for all times. All three curves with 25, 50, and 100 mM glucose are very close to each other due to the low λ_glu_ value compared to the total glucose concentration. Thus, it is unsurprising that model and data match well for the 50 mM curve, too.

Our Langmuir ansatz for the reactant concentration is mathematically identical to the Michaelis–Menten kinetics, which is often used to model enzymatic reactions.^8,15,29^ However, our model includes not only the free enzyme dynamics of the Michaelis–Menten kinetics, but also other glucose-dependent effects such as the glucose transport through the membrane. Thus, we decided to fit λ_glu_ = (5.88 ± 0.15) mM to the data and not use a literature value for the Michaelis–Menten constant of GOx.

The experiments report for the reference system a glucose consumption of 59 mM out of the initial 100 mM. Our model curve for this system consumes only 10 mM glucose. Additional 49 mM gluconic acid in the solution would decrease the pH below 3. The question is what happens with the remaining 49 mM glucose? One possible explanation is that the regular shaking of the system leads to spatial effects which are not included in the model. Another hypothesis is that the measurement of the remaining glucose concentration was influenced by a spatial glucose gradient. It is also possible that the batches of glucose and GOx include an unknown buffer due to the manufacturing process. We can manually increase the buffer capacity of the buffer solution or the AC-membranes in the model. However, in this case we are not able to match the *c*_glu,0_ = 25 mM curve, because the pH converges to a higher value. This problem also applies to the upcoming curves in Figure 4b which use only 20 mM urea. We decided to keep our model as is and accept that the glucose consumption does not match the experimental measurements. In exchange, the model covers the effects of the different tunable input parameters, the time scales, and the final pH.

**Figure 4:**
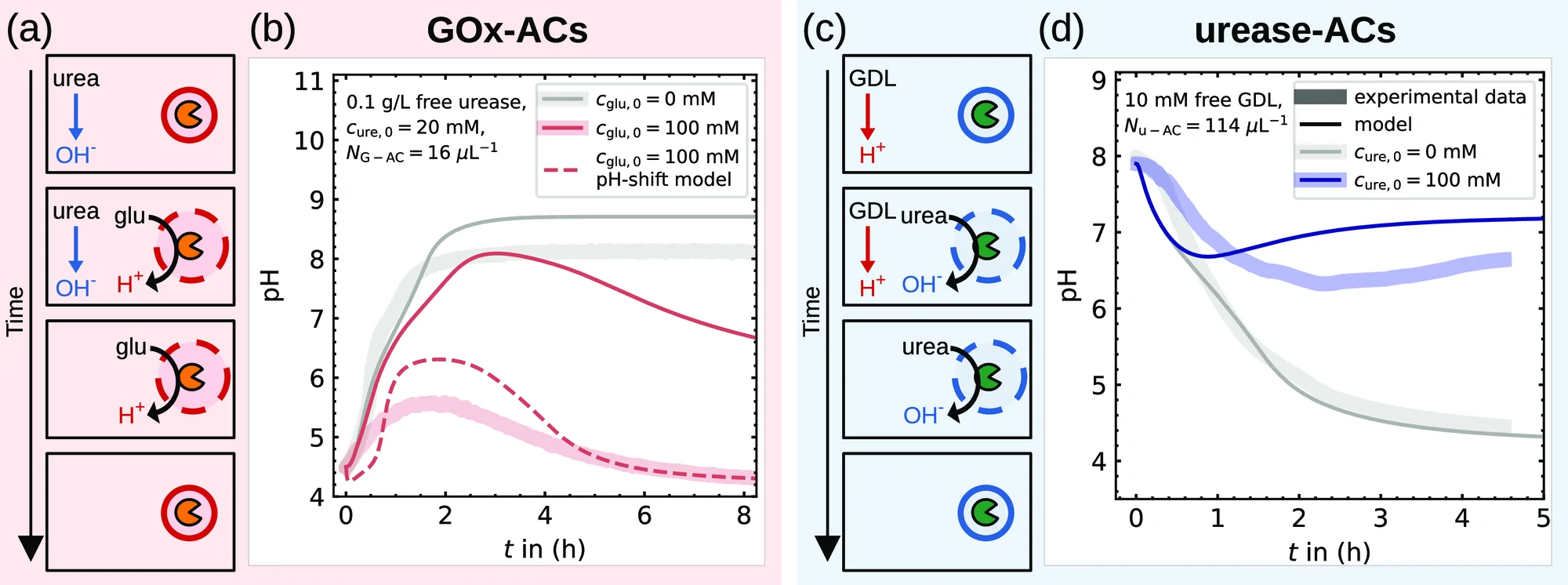
Protection mechanism of GOx-ACs and urease-ACs. (a) Urea is converted to ammonia until urea is depleted; thereby the pH increases. As a response the GOx-ACs produce gluconic acid which lowers the pH. (b) pH evolution with free urease, GOx-ACs, and without (gray) and with glucose (red). The wide lines correspond to the experiment,^11^ the thin lines to the model. The dashed line corresponds to the diagnostic pH-shifted model as described in the text. (c) GDL hydrolyzes into gluconic acid which lowers the pH. In response the urease-ACs produce ammonia which increases the pH. (d) pH evolution with free GDL, urease-ACs, and without (gray) and with urea (blue). The experimental data in (b,d) is reproduced from ref 11 used under CC-BY 4.0 license. Copyright 2024 Joshua Krehan et al. (http://creativecommons.org/licenses/by/4.0/).

Self-oscillations of the ACs are not possible within this model. The two-variable system with a single reaction rate is too simple for oscillations. Models which report self-oscillations have either a delayed response of the membrane^33,48^ or more variables.^31^ However, as experimentally shown, the pH can undergo cycles upon repeated addition of a base to the system.^11,20^ We can reproduce the experimental observations by repeatedly adding a strong base in our model (Figure S7).

#### Calibrating the Model for Urease-ACs

Urease-ACs work inversely compared to the GOx-ACs, as sketched in Figure 1b. The enzyme urease catalyzes the reaction

$$urea\left(ure\right)\to 2ammonia\left(amm\right)+{CO}_{2}(CO2).$$

The urease-ACs in the experiments have a poly­(2-(diethylamino)­ethyl methacrylate) shell, which becomes impermeable to urea above pH 7.3. Starting at pH 4, the urease-ACs convert urea into the base ammonia which in turn increases pH. The reaction stops when pH 7.3 is reached, because the membranes are impermeable to urea (see inset in Figure 1b).

We calibrate a model for the urease-ACs following the procedure for the GOx-ACs.^8,11,15,21,46,51–57^ We identify all participating acids and bases, look up the pH-dependent activity of urease,^15^ fit the membrane permeability to a reference curve, and fit the Langmuir constants to the data. The detailed model, procedure, fit parameters, and results are shown in Supporting Sections S1–S3 with Figures S1–S4. To obtain better fitting results, we reduce the buffer solution capacity to 5 mM and decrease the reaction rate within the first hour to account for initial effects in the experiments. But the model would still describe the experimental data even without the two model modifications. In total, the results describe the experimental observations. The fitting results for the Langmuir constants are slightly less accurate compared to the GOx-ACs; particularly, the model does not fully reproduce the final pH for different urea concentrations.

### Qualitative Validation Against the Protection Mechanism of ACs

#### Protection Mechanism of GOx-ACs

The GOx-ACs are able to automatically “protect” a low pH value against external perturbations in the form of a base, as shown in Figure 4a,b. In a system without glucose and initial pH 4.5, the addition of urea and free urease leads to an increase in pH due to the produced ammonia (gray line). If glucose and GOx-ACs are part of this system, the GOx-ACs respond to the rise in pH by becoming permeable. Thus, gluconic acid is produced which antagonizes the ammonia. The pH decreases until the GOx-ACs become again impermeable (red line).

We aim to validate the assumed feedback principles in the model by modeling the protection mechanism. To represent the external perturbation, we treat the free urease reaction with a modified version of the pH-dependent Michaelis–Menten rate by Ridgway-Brown et al.^15^ as an auxiliary model (Supporting Section S4.1).

We find in Figure 4b that the GOx-AC system without glucose and with free urease, matches the important initial time scale of the pH increase in the experiment. The same holds for free urease without GOx-ACs (Figure S4). Further, the model qualitatively predicts the experimentally observed protection mechanism and therefore validates the used feedback principles. The large overshoot to pH 8 in the model curve is partially caused by the high sensitivity of the positive feedback in the free urease reaction,^21^ which has an increasing reaction rate until pH 7 (maximum in the activity curve *a*(pH)). In addition, the model cannot produce gluconic acid below pH 4.6, although experimentally measured in the first minutes (slope comparison of the experimental lines) and after 5 h (pH decreases below 4.6). We hypothesize a higher AC-internal pH compared to the bulk, which is caused by the initial pH calibration. We support our explanations by a diagnostic pH-shifted model curve in Figure 4b with a four times higher *k*_GOx_ and a pragmatically shifted internal pH by using *a*(pH + 0.5)*P*(pH + 0.5).

### Protection Mechanism of Urease-ACs

The urease-ACs can perform a protection mechanism analogous to the one of the GOx-ACs as shown in Figure 4c,d. To a high pH solution, 10 mM free GDL is added which spontaneously hydrolyses into gluconic acid. The urease-ACs respond to the decreasing pH by swelling and producing ammonia out of urea. Thus, the pH increases again after a dip at around 2 h. The perturbing GDL is included only as auxiliary validation module into the model; its rate law follows a standard pH-dependent hydrolysis form^53^ and is summarized in Supporting Section S4.2. The modeled protection mechanism (blue line in Figure 4d) captures the key behavior by predicting the experimentally observed pH minimum and the subsequent pH increase. Unlike the experiment, the modeled minimum occurs after 1 h (instead of roughly 2 h) and also the subsequent increase happens faster. Possibly, there is a short response time in the permeability change of the membranes.

### AC-Mixture of Different AC-Types

Thus, far, we modeled and discussed only systems which were already experimentally tested. However, the working models for GOx-ACs and urease-ACs enable us to make predictions for systems that have not yet been studied experimentally. In particular, we look at AC-mixtures of GOx-ACs and urease-ACs as sketched in Figure 5a. In the experiments they only briefly examined AC-mixtures without an in-depth analysis of the parameters.^11^ The interest lies in the antagonistic behavior of both AC-types, where urease-ACs push the pH upward while GOx-ACs pull it downward, resulting in a tug-of-war.

**Figure 5:**
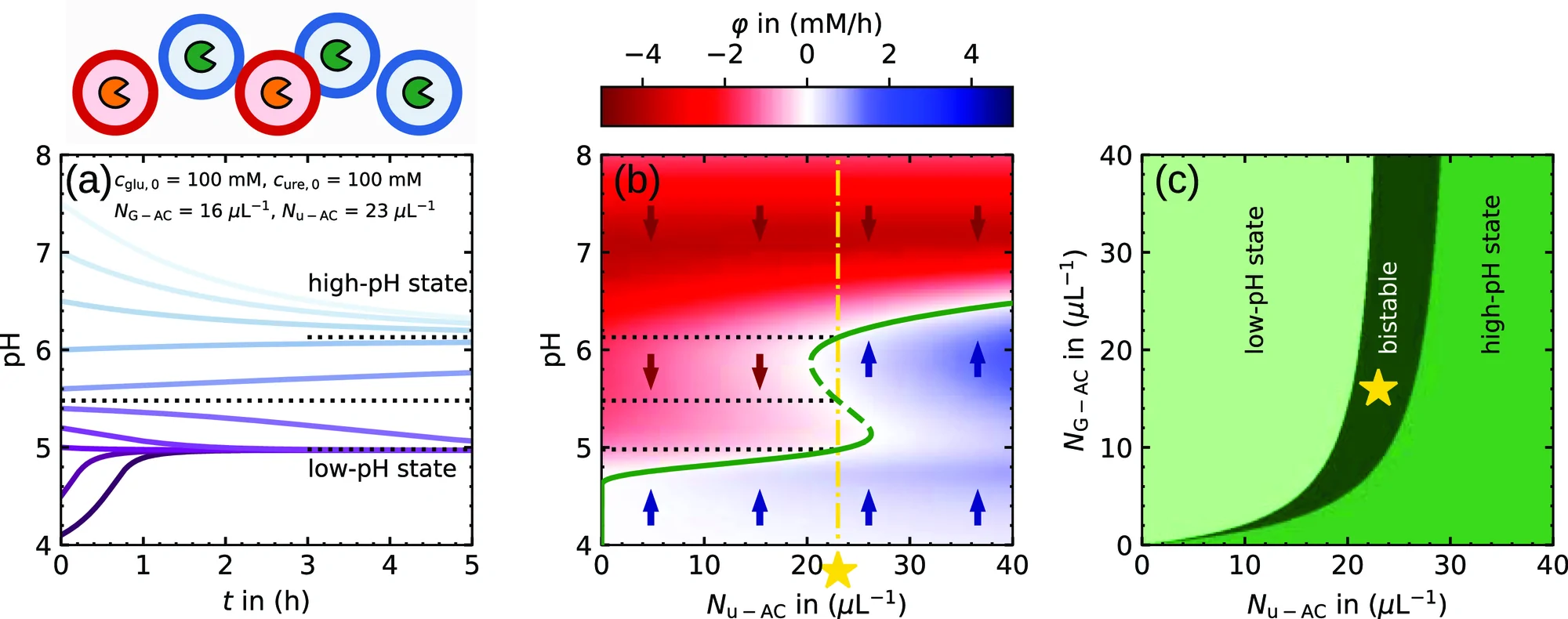
Short-time dynamics of AC-mixtures predicted with the model. (a) pH evolution for an AC-mixture (top sketch) for different initial pH (different colors; pH 4.1, 4.5, 5.0, 5.2, 5.35, 5.5, 6.0, 6.5, 7.0, 7.5). The used parameters are *c*_glu,0_ = 100 mM, *c*_ure,0_ = 100 mM, *N*_G‑AC_ = 16 μL^–1^, *N*_u‑AC_ = 23 μL^–1^, ρ_GOx_ = 10 g/L, ρ_urease_ = 1 g/L. The dotted lines represent the theoretically calculated pH for the low-pH state, unstable state, and high-pH state (see eq S8). (b) Stability map in the *N*_u‑AC_-pH-plane. In the red region with red arrows, the proton consumption rate φ of eq 4 is negative and the pH decreases, while it increases in the blue region with blue arrows. The green line shows the analytically calculated fixed points, which are stable in the solid part and unstable in the dashed part. The star and the dash-dotted line represent the AC concentration and fixed points of (a). (c) State diagram in the *N*_u‑AC_-*N*_G‑AC_-plane. The star indicates the parameters used in (a).

The model for the AC-mixtures builds upon the obtained models for GOx-ACs and urease-ACs. It consists of four rate equations, combining the two rate equations equations 1a, 1b of the GOx-system with the two rate equations of the urease-system (eq S1a, c). The pH is still calculated with eq 2 but now includes the acids and bases of both systems. We decided to use for the entire system the GOx-AC framework (10 mM buffer solution, no time delay term) to obtain a self-consistent model.

### Short-Time Dynamics

We first study the short-time dynamics happening within the first hours, where there is with *c*_glu,0_ = *c*_ure,0_ = 100 mM abundant fuel (glucose and urea). Figure 5a shows the short-time pH evolution of an AC-mixture (*N*_G‑AC_ = 16 μL^–1^, *N*_u‑AC_ = 23 μL^–1^). Depending on the initial pH the system converges to one of two stable fixed points in a bistable fashion. If the initial pH is below the unstable fixed point at pH 5.5, the system evolves toward the low-pH state (pH 5.0). If it is above, the system evolves toward the high-pH state (pH 6.1). The unstable fixed point arises from the nonlinear pH-dependencies of the enzyme activities and permeability terms.

The bistability does not exist for every set of parameters. Aiming for a quantitative analysis, we introduce a new observable φ to obtain the direction of pH change. Based on the charge balance eq 2 we define

4 $$\varphi \left(pH\right)=\frac{{ċ}_{amm}\left(pH\right)K_{b,amm}}{K_{b,amm}+K_{w}/\left[H^{+}\right]}-\frac{{ċ}_{ga}\left(pH\right)K_{a,ga}}{K_{a,ga}+\left[H^{+}\right]}-\frac{{ċ}_{CO2}\left(pH\right)K_{a,CO2}}{K_{a,CO2}+\left[H^{+}\right]},$$

which characterizes the proton consumption rate by the two reactions. For φ > 0, the pH increases; for φ < 0, it decreases; and φ = 0 marks a fixed point. Figure 5b shows φ in the *N*_u‑AC_-pH-plane. Although [φ] = mM/h has units of a rate in concentration, it does not directly reflect how fast the pH changes. The actual pH change rate depends in addition on the present acids and bases as well as on the current pH due to its logarithmic nature. However, the locations of the fixed points are not affected by these effects.

In Figure 5b we distinguish three different regions: For *N*_u‑AC_ < 20 μL^–1^ only the low-pH state exists. In this region the GOx-ACs outperform the urease-ACs. For *N*_u‑AC_ > 26 μL^–1^ it is reversed and only the high-pH state exists. Between these thresholds lies a bistable region with both stable states and one unstable fixed point; such a scenario is shown in Figure 5a. We support our findings by an analytical expression for the locations of the fixed points in Figure 5b (green line). To do this, we set φ = 0 in eq 4 and solve for *N*_u‑AC,fixedPoints_; see Supporting Section S6. Only the fixed points fulfilling *∂N*_u‑AC,fixedPoints_/∂pH > 0 are stable.

The results are generalized with a state diagram in Figure 5c which shows the *N*_u‑AC_-*N*_G‑AC_-plane. For the chosen parameters the bistable region always separates the low-pH state from the high-pH state. Moreover, we find that for every given GOx-AC concentration, the state can be tuned by varying the urease-AC concentration. Conversely, this is not possible by tuning the GOx-AC concentration for high urease-AC concentrations. The asymmetry originates from the different maximum reaction rates of both AC-types and the chosen enzyme concentrations. However, the latter can be modified, creating a four-dimensional state diagram to tune the system’s behavior.

### Long-Time Dynamics

After a long time, one or both fuels run out, which introduces a second, slower time scale into the dynamics. We see this exemplified in Figure 6a by starting at pH 5.5 with 300 mM glucose and four different urea concentrations between 75 mM and 150 mM. For short times, the pH evolution splits into the low-pH state for *c*_ure,0_ ≤ 100 mM and the high-pH state otherwise. The trajectories with low urea remain in the low-pH state and follow the fixed point line in Figure 6b. This figure presents the stability map under the constant high glucose concentration approximation *c*_glu_(*t*) = 300 mM. Eventually, the pH converges to 4.6, because urea runs out while glucose is still left (Figure 6c). This remaining glucose is converted into gluconic acid until the GOx-ACs become fully impermeable and therefore all chemical reactions stop.

**Figure 6:**
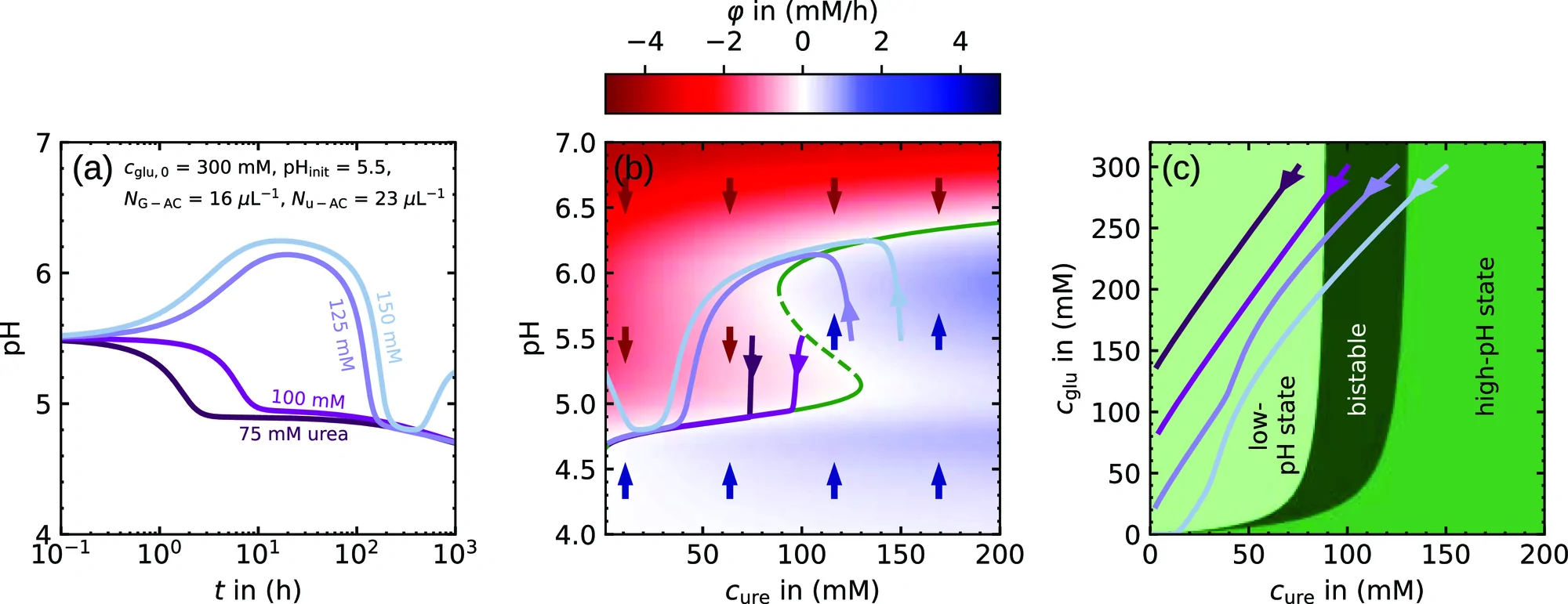
Long-time dynamics of AC-mixtures predicted with the model. (a) pH evolution for different initial urea concentrations (different colors) in a log-lin-plot. The parameters are *c*_glu,0_ = 300 mM, *N*_G‑AC_ = 16 μL^–1^, *N*_u‑AC_ = 23 μL^–1^, ρ_GOx_ = 10 g/L, ρ_urease_ = 1 g/L, and an initial pH of 5.5. (b) Stability map in the *c*_ure_-pH-plane with *c*_glu,0_ = 300 mM. In the red region with red arrows, the proton consumption rate φ of eq 4 is negative and the pH decreases, while it increases in the blue region with dark blue arrows. The green line shows the analytically calculated fixed points (see eq S9), which are stable in the solid part and unstable in the dashed part. The other colored lines show the trajectories from (a). (c) State diagram in the *c*_ure_-*c*_glu_-plane. The colored lines show the trajectories from (a).

The trajectories with high initial urea concentrations stay for tens of hours in the high-pH state while glucose and urea are consumed, see Figure 6a. When the urea concentration decreases, the reaction rate of the urease-ACs slows down. As a consequence, the GOx-ACs take the lead, the high-pH state vanishes, and the trajectories run in the low-pH state as shown in Figure 6b. The duration and pH within the transient high-pH state increase with increasing initial urea concentration. The trajectories need roughly 200 h to reach the low-pH fixed point line because the time scale of urea consumption is slower than the time scale of pH change. At longer times Figure 6c shows that the 150 mM-urea trajectory runs first out of glucose, which breaks the constant high glucose approximation in Figure 6b. After 400 h the pH increases again until urea is also depleted and the final pH is reached. The final pH is always predetermined by the initial pH and the initial fuel concentrations. It can be calculated by assuming that glucose and urea are fully converted into their respective acids and bases. The only constraint is that the final pH is between 4.6 and 7.3, which are the closing pH values of the ACs (*P*_GOx_(pH < 4.6) = 0, *P*_urease_(pH > 7.3) = 0).

The full observed long-time dynamics happens on a time scale of several hundreds of hours, during which enzymes could degrade. It is possible to accelerate the dynamics by 1 order of magnitude by adjusting the input parameters (less GOx-ACs, more urease-ACs, lower initial fuel concentrations, no buffer solution).

The finding of a late pH increase when one fuel runs out is not new, but has been observed in experiments of enzymatic reaction networks with urease-GOx and urease–esterase reaction networks.^19^ Similar behaviors occur when the enzymes are spatially separated and an initial distance of ester and esterase leads to a delayed pH decrease.^18^ Also bistability in the pH value has been found in enzymatic reaction networks with urease and esterase.^19^ In accordance with experiments of AC-mixtures, oscillations are not possible without the repeated addition of acids or bases.^12,20^

## Conclusions

In summary, we have presented a reaction model for acid- or base-producing ACs with pH-responsive membranes. The model tracks the bulk substrate and product concentrations and incorporates the pH-dependencies of the enzyme activity and membrane permeability. Additional effects, such as the dependence on AC concentration, are captured with a Langmuir-type term. We calibrated the model to recent GOx-AC and urease-AC experiments.^11^ The model covers the main trends of the experiment and enables us to make predictions for other parameter choices. We extended the study to mixtures of GOx-ACs and urease-ACs by combining the two fitted models. The antagonistic reaction products lead to three different regimes: a stable low-pH state, a stable high-pH state, and bistability of both states. The bistable pH states imply hysteresis in the collective behavior and enable switchable chemical modules. Finally, we analyzed the long-time dynamics of the AC-mixtures. The results reveal fuel-controlled transient states, with tunable lifetimes and final pH. We are confident that these findings can guide future experiments in the realm of ACs. In particular, because the studied parameters are easily tunable in experiments, such as AC concentrations, enzyme concentrations, and fuel concentrations.

The present model performs well for small systems or stirring experiments. However, in large systems the diffusion time scale needs to be addressed. As shown for different systems, pH fronts can occur for local acid/base productions.^8,13,58^ In combination with metamaterials this enables spatiotemporal control of hydrogel stiffening.^59,60^ Combining spatiotemporal diffusion of chemicals with antagonistic enzyme-powered ACs could lead to more enhanced feedback mechanisms. It aligns with the upcoming direction of networks of communicating ACs,^7^ which we believe will be important for the design of “smart” (meta)­materials with internal capabilities such as oscillations, adaptive responses, and division of labor.

Importantly, the observed chemical bistability suggests possible functionality. Since the low- and high-pH states are both stable over a finite parameter range, AC-mixtures could act as chemically switchable modules with memory: a transient perturbation or change in composition may select one of two persistent pH states. Such states could be coupled to pH-sensitive downstream reactions, membrane permeability, cargo release, hydrogel swelling, or matrix stiffening. In this sense, antagonistic AC-mixtures provide a minimal design principle for chemically programmable soft materials, in which pH is not merely regulated but used as a bistable internal control variable.

## Supplementary Material



## Notes

Table with model parameters. Model setup for urease-AC systems. Model details for both AC-types about buffer solution, buffer capacity of membranes, permeability term, free urease, and free GDL. Additional analysis about the effect of the GOx-AC concentration and repeated acid/base addition. Analytical solutions for the steady states in AC-mixtures (PDF)
