# Supplementary material for: Chemical Feedback and Collective pH-Dynamics in Enzyme-Powered Artificial Cells
Source: J Phys Chem B. 2026 Sep 21;130(39):10269–78. doi: 10.1021/acs.jpcb.6c03926 (PMC13637022; doi:10.1021/acs.jpcb.6c03926)
Supplement: Supplementary file 1 [file jp6c03926_si_001.pdf]

Supporting Information: Chemical Feedback and Collective pH-Dynamics in Enzyme-Powered Artificial Cells

Nils Göth[1] and Joachim Dzubiella*[1,2]

[1]Applied Theoretical Physics–Computational Physics, Physikalisches Institut, Albert-Ludwigs-Universität Freiburg, D-79104 Freiburg, Germany

[2]Cluster of Excellence livMatS @ FIT - Freiburg Center for Interactive Materials and Bioinspired Technologies, Albert-Ludwigs-Universität Freiburg, D-79110 Freiburg, Germany

*Email: joachim.dzubiella@physik.uni-freiburg.de

Contents

S1 Table with Parameters **S2**

S2 Urease-AC Model **S3**

S2.1 pH Calculation . . . S3

S2.2 Enzyme Activity . . . S4

S2.3 Permeability Term . . . S4

S2.4 Calibrating the Effects of Tunable Input Parameters . . . S6

S3 Model Details for GOx-ACs and Urease-ACs **S8**

S3.1 DPBS Buffer Solution . . . S8

S3.2 Buffer Capacity of Membranes . . . S8

S3.3 Permeability Term . . . S8

S3.3.1 Relation between Permeability Term and AC Diameter . . . S9

S4 Model Details for Free Enzymes **S10**

S4.1 Free Urease in the GOx-AC System . . . S10

S4.2 Free GDL in the Urease-AC System . . . S10

S5 Additional Analysis **S13**

S5.1 Required Time for the pH Decrease for Different GOx-AC Concentrations . . . S13

S5.2 Repeated Acid/Base Addition . . . S14

S6 Analytical Solutions for Fixed Points in AC-Mixtures **S16**

S6.1 Analytical Solution Solved for the Urease-AC Concentration . . . S16

S6.2 Analytical Solution Solved for the Urea Concentration . . . S16

S1 Table with Parameters

Table S1: **Model parameters for the GOx-AC system (left) and the Urease-AC system (right).**

**GOx-AC system**			**urease-AC system**		
Parameter	Value	Reference	Parameter	Value	Reference
		Enzymatic reaction rate constants			
k_{GOx} [mM/h]	8.3	fit	k_{urease} [mM/h]	6.9	fit
		Reference parameters			
$N^{\text{r}}_{\text{G-AC}}$ [μL^{-1}]	16		$N^{\text{r}}_{\text{u-AC}}$ [μL^{-1}]	114	
$\rho^{\text{r}}_{\text{GOx}}$ [g/L]	10		$\rho^{\text{r}}_{\text{urease}}$ [g/L]	1	
$c^{\text{r}}_{\text{glu}}$ [mM]	100		$c^{\text{r}}_{\text{ure}}$ [mM]	100	
		Langmuir constants			
$\lambda_{\text{G-AC}}$ [μL^{-1}]	7.91	fit	$\lambda_{\text{u-AC}}$ [μL^{-1}]	$1 \cdot 10^{8}$	fit
λ_{GOx} [g/L]	1.139	fit	λ_{urease} [g/L]	$1 \cdot 10^{-6}$	fit
λ_{glu} [mM]	5.69	fit	λ_{ure} [mM]	210	fit
		Enzyme activity			
$K_{\text{es1,GOx}}$ [M]	$7.94 \cdot 10^{-5}$	ref [1]	$K_{\text{es1,urease}}$ [M]	$5 \cdot 10^{-6}$	ref [2]
$K_{\text{es2,GOx}}$ [M]	$3.98 \cdot 10^{-8}$	ref [1]	$K_{\text{es2,urease}}$ [M]	$2 \cdot 10^{-9}$	ref [2]
		Membrane permeability			
A_{GOx}	3.7	fit	A_{urease}	0	fit/fixed
$y_{1,\text{GOx}}$	4.6	fit/fixed	$y_{1,\text{urease}}$	7.3	fit/fixed
$y_{2,\text{GOx}}$	5.73	fit	$y_{2,\text{urease}}$	–	
$d_{1,\text{GOx}}$	3.07	fit	$d_{1,\text{urease}}$	−2.22	fit
$d_{2,\text{GOx}}$	0.86	fit	$d_{2,\text{urease}}$	–	
		Acid/base dissociation constants			
$\text{p}K_{\text{a,ga}}$	3.86		$\text{p}K_{\text{a,CO2}}$	6.35	
			$\text{p}K_{\text{b,amm}}$	4.75	
$\text{p}K_{\text{w}}$	14		$\text{p}K_{\text{w}}$	14	
$\text{p}K_{\text{a,PMAA}}$	6.25	ref [3]	$\text{p}K_{\text{b,PDEAEMA}}$	6.9	ref [4, 5]
$\text{p}K_{\text{a,DPBS}}$	6.86		$\text{p}K_{\text{a,DPBS}}$	6.86	
$\text{p}K_{\text{b,DPBS}}$	7.17		$\text{p}K_{\text{b,DPBS}}$	7.17	
		Buffer concentrations			
n_{carboxyl} [mol/AC]	$1.1 \cdot 10^{-10}$	Section S3.2	n_{amine} [mol/AC]	$2.7 \cdot 10^{-11}$	Section S3.2
$c_{\text{a,DPBS}}$ [mM]	1.5	experiment	$c_{\text{a,DPBS}}$ [mM]	0.75	modified exp.
$c_{\text{b,DPBS}}$ [mM]	8.1	experiment	$c_{\text{b,DPBS}}$ [mM]	4.05	modified exp.
	Free urease reaction			*Free GDL hydrolysis*	
$k^{\text{free}}_{\text{urease}}$ [1/h]	0.0141	fit	$k^{\text{free}}_{\text{GDL}}$	2.42	fit
K_{M} [mM]	3	ref [2]	K_{δ}	3.68	ref [6]
			$K_{\text{a,GDL}}$ [mol^{-1}L]	$2.40 \cdot 10^{-4}$	ref [6]
			$k_{\text{H}_2\text{O}}$ [h^{-1}]	0.165	ref [6]
			k_{OH^-} [$\text{h}^{-1}\text{Lmol}^{-1}$]	$9.94 \cdot 10^{6}$	ref [6]

S2 Urease-AC Model

The approach for the urease-AC model is analogous to the GOx-AC model. Therefore, we follow the same procedure for the urease-AC model as for the GOx-AC model in the main manuscript: general system, pH calculation, enzyme activity, membrane permeability, and the effects of tunable input parameters.

The main chemical reaction in the urease-ACs is catalyzed by the enzyme urease

$$\text{urea (ure)} \rightarrow 2\ \text{ammonia (amm)} + \text{carbon dioxide (CO2)}.$$

The reaction converts one urea molecule into two ammonia molecules (base) and one CO_2 molecule (acid). Because more base than acid is produced, the reaction products result in a net basic effect.

We model the system with three differential equations for the bulk concentrations of urea c_{ure}, ammonia c_{amm}, and carbon dioxide c_{CO2}. These three differential equations are coupled via the reaction rate yielding

$$\dot{c}_{\text{amm}} = -2\dot{c}_{\text{ure}}, \tag{S1a}$$

$$\dot{c}_{\text{CO2}} = -\dot{c}_{\text{ure}}, \tag{S1b}$$

$$\dot{c}_{\text{ure}} = -k_{\text{urease}} T(t) a(\text{pH}) P(\text{pH}) B_{\text{ref}} \frac{N_{\text{u-AC}}}{\lambda_{\text{u-AC}} + N_{\text{u-AC}}} \frac{\rho_{\text{urease}}}{\lambda_{\text{urease}} + \rho_{\text{urease}}} \frac{c_{\text{ure}}}{\lambda_{\text{ure}} + c_{\text{ure}}}, \tag{S1c}$$

with $B_{\text{ref}} = \frac{\lambda_{\text{u-AC}} + N^{\text{r}}_{\text{u-AC}}}{N^{\text{r}}_{\text{u-AC}}} \frac{\lambda_{\text{urease}} + \rho^{\text{r}}_{\text{urease}}}{\rho^{\text{r}}_{\text{urease}}} \frac{\lambda_{\text{ure}} + c^{\text{r}}_{\text{ure}}}{c^{\text{r}}_{\text{ure}}}$. The reference system has the parameters $N^{\text{r}}_{\text{u-AC}} = 114\ \mu\text{L}^{-1}$, $\rho^{\text{r}}_{\text{urease}} = 1$ g/L, and $c^{\text{r}}_{\text{ure}} = 100$ mM.

In our setup, the model equations reduce to an effective two-variable system, because the equations (S1a) and (S1b) are coupled via the relation $c_{\text{amm}} = 2c_{\text{CO2}}$ for initial conditions $c_{\text{amm},0} = 2c_{\text{CO2},0}$. The latter is true, because there is neither ammonia nor CO_2 in the bulk solution at $t = 0$.

The additional term $T(t)$ is of the form

$$T(t) = \min(1,\ t/(1\ \text{h})) \tag{S2}$$

and increases linearly from 0 to 1 within the first hour and then stays at 1. It is added as a multiplicative factor to the reaction rate because the experimentally observed pH evolution in urease systems shows initially a slow increase (see Figure S2a for urease-ACs (gray line) and free urease (green line)). This slow increase cannot be explained by the bell-shaped activity curve of urease. It also stands in contrast to other experimental observations with free urease [7]. Thus, we assume that it comes from an intrinsic delay of the experimental setup. To account for it, we use the simple, pragmatic delay term in eq (S1c).

S2.1 pH Calculation

We calculate the pH by numerically solving the charge balance equation (2) analogous to the procedure for the GOx-ACs. We use the base and acid dissociation constants of ammonia and CO_2, see Tab. S1.

While the experiments use 10 mM DPBS buffer solution, we use only 5 mM in the model. The reason lies in the experimental data of 10 mM free GDL without ACs in Figure S4b. The 10 mM GDL is fully or partially converted into gluconic acid yielding pH 4. With 10 mM DPBS buffer solution, the gluconic acid would be nearly entirely buffered and the system could not reach a low pH. Therefore, we decided to reduce the concentration of the buffer solution in the model to 5 mM. The resulting model curve is shown in Figure S4b. Model details about the GDL reaction are given in Section S4.2.

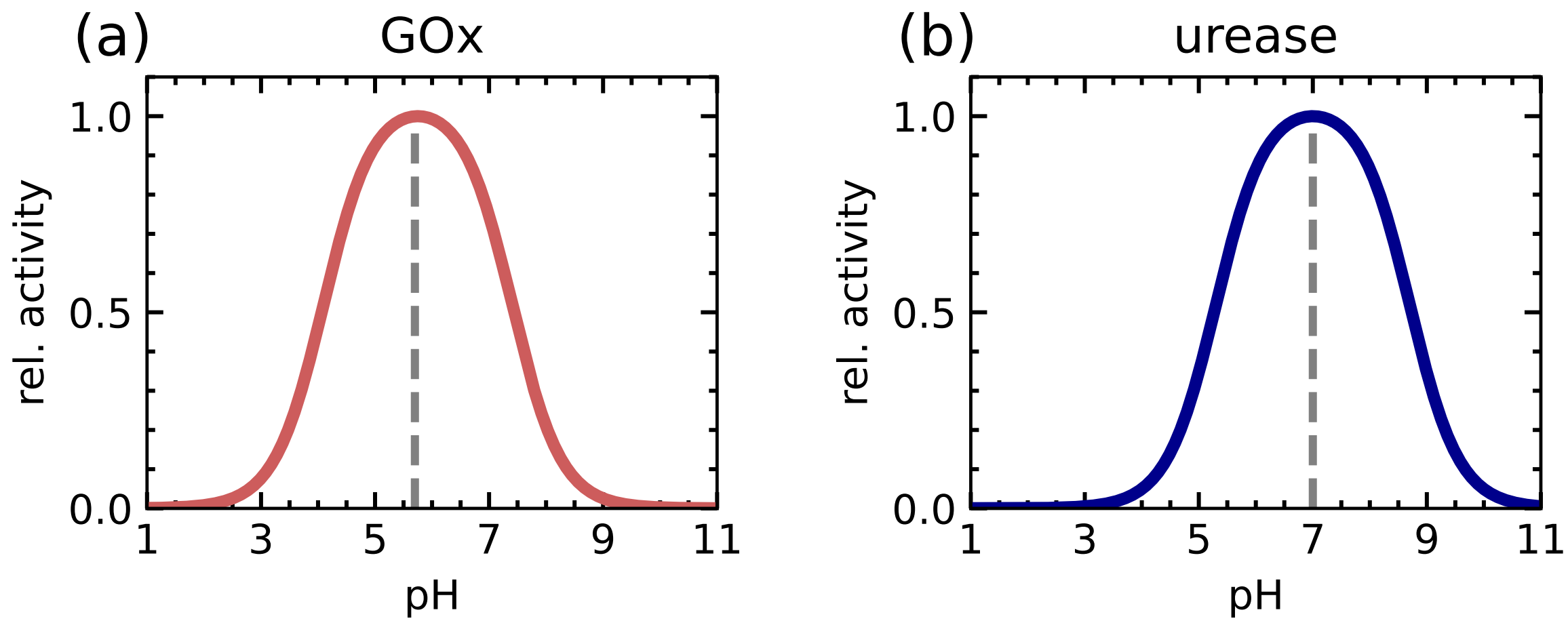

Figure S1: **Activity curves of the enzymes.** Normalized bell-shaped activity curves $a(\mathrm{pH})$ of **(a)** GOx and **(b)** urease as a function of pH (cf. eq (3)). The dashed lines label the maximum at pH 5.7 for GOx and 7.0 for urease.

The buffer effect of the urease-AC membranes is based on the amine groups in the poly(2-(diethylamino) ethyl methacrylate) (PDEAEMA) polymer network. We treat the amine groups as a normal weak base, analogous to the treatment of the carboxyl groups in the GOx-ACs membranes. Based on literature values of free and star-shaped PDEAEMA polymers, we estimate $\mathrm{p}K_{\mathrm{b,PDEAEMA}} = 6.9$ [4, 5]. We calculate a concentration of $n_{\mathrm{PDEAEMA}} = 2.8 \cdot 10^{-11}$ mol/AC from the geometry and membrane composition; for the calculation see Section S3.2. Converted to 114 ACs/μL, this value corresponds to a buffer capacity of 3.1 mM.

S2.2 Enzyme Activity

For the pH-dependent enzyme activity, we use the expression of eq (3)

$$a([\mathrm{H}^+]) = \left(1 + \frac{K_{\mathrm{es2}}}{[\mathrm{H}^+]} + \frac{[\mathrm{H}^+]}{K_{\mathrm{es1}}}\right)^{-1}. \tag{S3}$$

Figures S1a,b show the pH-dependent activity curves of GOx and urease. In experiments, the activity curve of urease, as for GOx, shows bell-shaped behavior [8, 9], suggesting roughly $K_{\mathrm{es1,urease}} = 5 \cdot 10^{-6}$ M and $K_{\mathrm{es2,urease}} = 2 \cdot 10^{-9}$ M for the ionization constants [8]. We use these values in eq (3), resulting in a maximum at pH 7.0. Existing models for the urea-urease reaction also use these values [10, 11].

The ansatz of eq (3) for GOx and urease ignores minor changes to the activity, such as the inhibition from phosphate buffer solutions [12, 13] or the temperature dependence [9].

S2.3 Permeability Term

We extract the permeability term of the urease-ACs from the experimental curve with the reference values $N^{\mathrm{r}}_{\text{u-AC}} = 114\ \mu\mathrm{L}^{-1}$, $\rho^{\mathrm{r}}_{\mathrm{urease}} = 1$ g/L, and $c^{\mathrm{r}}_{\mathrm{ure,0}} = 100$ mM. The experimental pH evolution is shown in Figure S2a. From the experimental data, we calculate with eq (2) and $c_{\mathrm{amm}}(t) = 2c_{\mathrm{CO2}}(t)$ the ammonia concentration and its time derivative $\dot{c}_{\mathrm{amm}}(t)$. For the used reference values, the $\lambda_{\text{u-AC}}$- and $\lambda_{\mathrm{urease}}$-term cancel with their counterparts in B_{ref}. The initial urea concentration of $c_{\mathrm{ure,0}} = 100$ mM is high compared

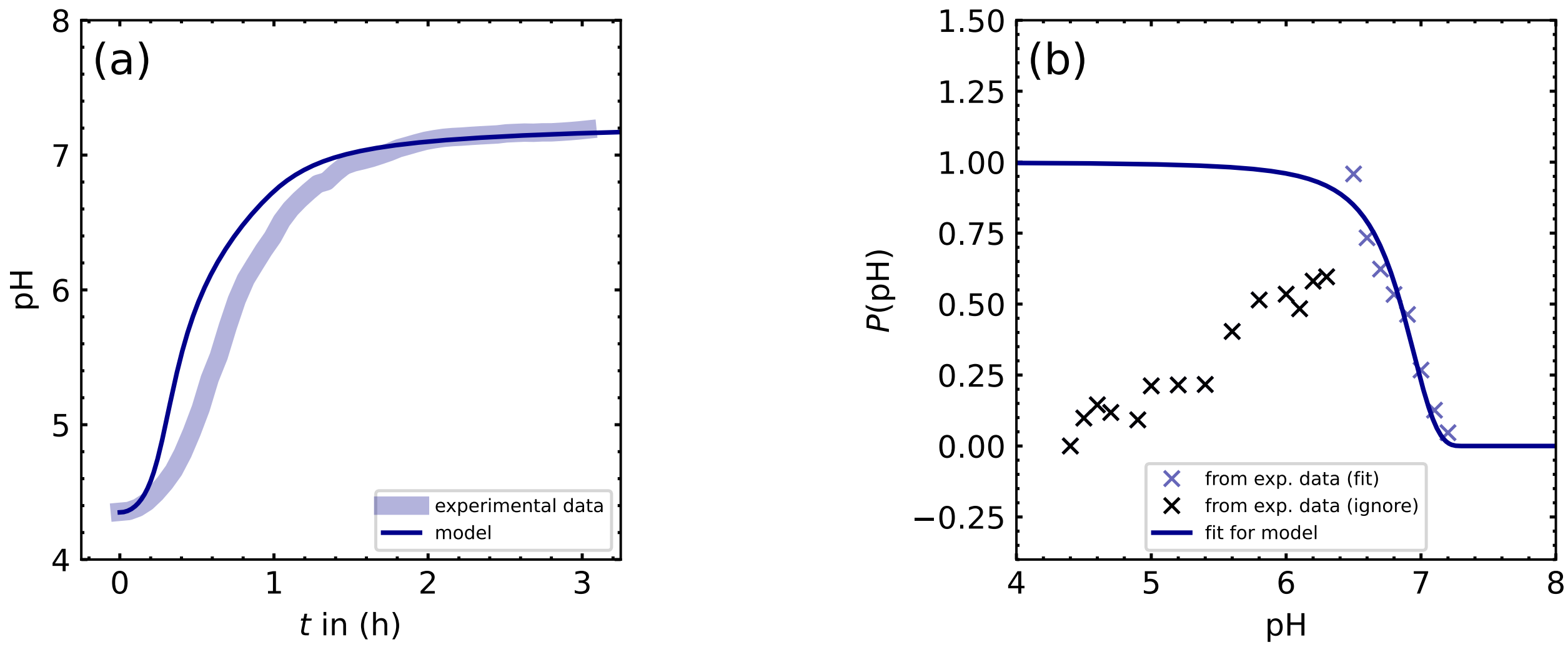

Figure S2: **Determination of the permeability term of the urease-AC membranes.** **(a)** Time evolution of pH in the experiments [14] (light blue) and in the model (dark blue). **(b)** Permeability term as a function of pH. The crosses are obtained from the experimental data using the model eq (1b). The solid line is a fit to the blue crosses. The black crosses (pH < 6.4) are excluded because they correspond to the initial phase of the experiment. The fitted $P(\mathrm{pH})$ is put back into the model, which then results in the modeled pH evolution in (a). The experimental data in (a) is reproduced from ref [14] used under CC-BY 4.0 license. Copyright 2024 Joshua Krehan et al. (http://creativecommons.org/licenses/by/4.0/).

to λ_{ure} and the urea consumed, so we cancel the λ_{ure}-term with its counterpart in B_{ref}, too. With $P \propto \dot{c}_{\mathrm{amm}}/(2a(\mathrm{pH}))$, we obtain the unnormalized permeability data shown as crosses in Figure S2b. The prefactor 1/2 comes from the urease reaction, which produces two ammonia molecules from one urea molecule; see eq (S1a). The data below pH 6.5 are part of the slow pH increase within the first hour, which includes the delay term $T(t) < 1$. We observe this slow pH increase also in the experimental P-data, where it is lower than the maximum at pH 6.4. Thus, we decided to fit $P(\mathrm{pH})$ only to the data for pH > 6.4. In this range we observe a single sigmoidal decrease. We apply the condition $P(\mathrm{pH} = 7.3) = 0$ due to experimental observations (Figure S2a) and the normalization condition $P(\mathrm{pH} \to 0) = 1$. The latter allows us to fit the prefactor of the reaction rate in eq (S1c), yielding $k_{\mathrm{urease}} = (0.0141 \pm 0.0012)/\mathrm{h}$. The exact form and fit parameters of $P(\mathrm{pH})$ are given in Section S3.3.

The single sigmoidal form of $P(\mathrm{pH})$ is different from the permeability term of the GOx-ACs, which shows a double sigmoidal form; see Figure 2b. For both AC types, one sigmoidal decrease can be assigned to the collapse of the AC. We explain the second decrease in $P_{\text{G-AC}}(\mathrm{pH})$ with conformational changes in the membrane's polymer network. These conformational changes lead to a fully impermeable membrane. For urease-ACs the membranes are already fully impermeable to urea after the initial decrease. Further conformational changes in the polymer network at higher pH are possible, which would affect the permeability of other, smaller molecules.

Inserting the fitted $P(\mathrm{pH})$ back into the model equations yields the modeled pH evolution shown in Figure S2a. We observe that the fit describes the general behavior of the pH increase well. However, there is a difference in the timing of the pH increase. This time difference highlights a crucial difficulty in fitting the model to the urease-AC data. The experimental data curves are shifted in time such that the initial pH of the shown curves match, to best compare the differences. This can be seen by comparing the dark blue lines in Figs. S2a, S3a-c, which all show the identical data, but are shifted in time by up to 30 min. Combined

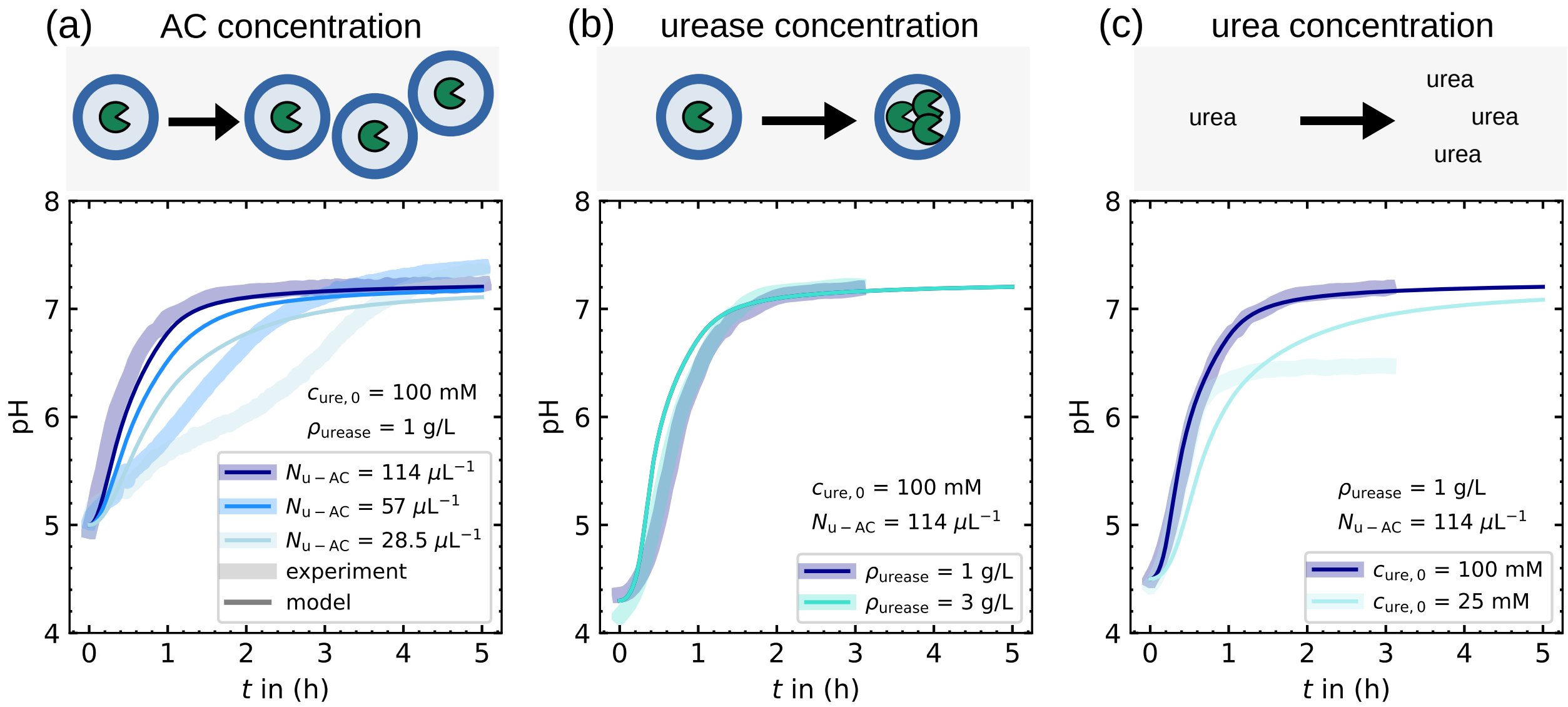

Figure S3: **pH evolution for urease-ACs and different input parameters.** Variation of **(a)** AC concentration $N_{\text{u-AC}}$, **(b)** urease concentration ρ_{urease}, and **(c)** initial urea concentration $c_{\text{ure},0}$. The thick lines correspond to the experimental data [14], the thin lines to the model. The reference system used to fit $P(\text{pH})$ is always shown in dark blue. The parameter λ_{ure} is fitted to the data with $c_{\text{ure},0} = 25$ mM. The experimental data is reproduced from ref [14] used under CC-BY 4.0 license. Copyright 2024 Joshua Krehan et al. (http://creativecommons.org/licenses/by/4.0/).

with the initial timing effect of $T(t)$, this results in different modeled curves in Figs. S2a, S3a-c even though they correspond to the same parameter set. Nevertheless, independent of time shift, the qualitative trends of the model match very well with the experimental data, and enable a qualitative model for the urease-ACs.

S2.4 Calibrating the Effects of Tunable Input Parameters

The effects of the AC concentration $N_{\text{u-AC}}$, the enzyme concentration ρ_{urease}, and the urea concentration $c_{\text{ure}}(t)$ are modeled via a Langmuir-type ansatz; analogous to the GOx-ACs. The remaining free fit parameter is in each case λ_i.

Figure S3a shows the pH evolution for three different AC concentrations. We observe in the comparison between the experimental lines with 57 ACs/μL and 114 ACs/μL that doubling $N_{\text{u-AC}}$ leads to more than double the speed in pH increase, which would mean a super-linear scaling. We believe that this is an effect of shifting the experimental curves in time as discussed in Section S2.3. The model fits best with $\lambda_{\text{u-AC}} \to \infty$ which implies that the reaction rate is proportional to $N_{\text{u-AC}}$. For numerical reasons we use the finite value $\lambda_{\text{u-AC}} = 10^8\ \mu\text{L}^{-1}$. This covers the qualitative behavior of the experiments in Figure S3a, but shows a slight difference in the time scale of pH increase.

Figure S3b shows the pH evolution for two different urease concentrations. We observe in the experimental data that the pH evolution is identical for 1 g/L and 3 g/L urease. This means that $\lambda_{\text{urease}} \ll 1$ g/L; a more specific estimation is not possible. We use $\lambda_{\text{urease}} = 10^{-6}$ g/L which means that the reaction rate is independent of the urease concentration.

Figure S3c shows the pH evolution for two different initial urea concentrations. The experimental curves converge to pH 7.2 for 100 mM urea and pH 6.4 for 25 mM urea. This is supported by the measured 59 mM consumed urea in the reference setup. Also previous experiments of urea-urease reactions with a

buffer solution have shown that the final pH can depend on the initial urea concentration [7]. The difference in Figure S3c would imply a buffer concentration of the order of 25 mM. With 5 mM buffer solution and 3.1 mM buffer capacity of the AC membranes the value is significantly lower than required. However, the free GDL curves in Figure S4b do not allow higher buffer concentrations, as discussed in Section S2.1. Consequently, our model is not able to accurately predict the final pH value for varying urea concentrations, cf. Figure S3c. Nevertheless, we fit the Langmuir constant to the experimental curve with 25 mM and obtain $\lambda_{\mathrm{ure}} = (210 \pm 90)$ mM.

S3 Model Details for GOx-ACs and Urease-ACs

S3.1 DPBS Buffer Solution

A DPBS buffer solution (Dulbecco's Phosphate Buffered Saline) is added during the experiments to stabilize the initial pH. It consists of 0.2 g/L KCl, 0.2 g/L KH_2PO_4, 8.0 g/L NaCl, and 1.15 g/L Na_2HPO_4 [15]. The primary buffering agents are KH_2PO_4 with the acid dissociation constant $\mathrm{p}K_{\mathrm{a,DPBS}} = 6.86$ and Na_2HPO_4 with $\mathrm{p}K_{\mathrm{b,DPBS}} = 7.17$. For a 10 mM buffer solution, the resulting molar concentrations are $c_{\mathrm{KH_2PO_4}} =$ 1.5 mM and $c_{\mathrm{Na_2HPO_4}} = 8.1$ mM. If less buffer solution is used, both molar concentrations are scaled accordingly.

S3.2 Buffer Capacity of Membranes

The membranes of the PMAA-ACs contain carboxyl groups which have an acidic behavior. Analogously, the PDEAEMA-ACs have amine groups which act as a base. In the following, we estimate the number of carboxyl (amine) groups per AC to treat the AC buffer effect as a weak acid/base.

In the collapsed state, the PMAA-ACs have a diameter of 55.3 μm and a membrane thickness of 1.45 μm. This results in a membrane volume of $V_{\mathrm{mem}} = 1.4 \cdot 10^{-8}$ cm^3/AC. Together with a PMAA density of $\rho_{\mathrm{mem}} = 1.035$ g/cm^3 (monomer density), a molecular mass of $m_{\mathrm{PMAA}} = 542.6$ g/mol per repeating unit after hydrolysis, and four carboxyl groups per repeating unit [16], we obtain for the number of carboxyl groups per AC $n_{\mathrm{cg}} = 4V_{\mathrm{mem}}\rho_{\mathrm{mem}}/m_{\mathrm{PMAA}} = 1.1 \cdot 10^{-10}$mol/AC. For 16 ACs/$\mu$L this yields a buffer capacity of 1.7 mM.

The analogous calculation is done for the PDEAEMA-ACs with a diameter of 43.3 μm, a membrane thickness of 1.05 μm (collapsed state), a PDEAEMA density of $\rho_{\mathrm{mem}} = 0.922$ g/cm^3 (monomer density) and a molecular mass of $m_{\mathrm{PDEAEMA}} = 185.3$ g/mol. This results in a number of amine groups per AC $n_{\mathrm{ag}} = 2.7 \cdot 10^{-11}$mol/AC. For 114 ACs/$\mu$L this corresponds to a buffer capacity of 3.1 mM.

The calculated buffer capacities are supported by a model-experiment comparison. Figure S4a shows the pH evolution of free urease without and with GOx-ACs. Thus, the two systems differ solely by the buffer effect of the ACs. The final pH of the two model curves differs by 0.1, which is comparable to the two experimental curves. This supports the calculated buffer capacity, even though deviations of 50% or more are still possible. Similarly, Figure S4b shows the pH evolution of free GDL without and with urease-ACs. Here, the final pH values of the two model curves differ by 0.6 which is similar to the final pH difference of the two experimental curves of 0.4. The discrepancy in the final pH between model and experiment is not important as mentioned Sections S4.1 and S4.2.

S3.3 Permeability Term

The observed permeability term for GOx-AC membranes in Figure 2b shows a two-step behavior. We fit to the data a double sigmoidal function of the form

$$P(\mathrm{pH}) = \frac{1}{A+1}\frac{\Theta(d_1(\mathrm{pH}-y_1))\,[d_1(\mathrm{pH}-y_1)]^3}{1+\Theta(d_1(\mathrm{pH}-y_1))\,[d_1(\mathrm{pH}-y_1)]^3} + \frac{A}{A+1}\frac{\Theta(d_2(\mathrm{pH}-y_2))\,[d_2(\mathrm{pH}-y_2)]^3}{1+\Theta(d_2(\mathrm{pH}-y_2))\,[d_2(\mathrm{pH}-y_2)]^3}. \quad \text{(S4)}$$

Equation (S4) consists of two sigmoidal terms which are weighted by A. Each sigmoidal term has the general shape $x^3/(1+x^3)$. The sigmoidal terms start at $y_{1,2}$ and have a steepness of $d_{1,2}$. In combination with the Heaviside step functions $\Theta(\dots)$, this guarantees that the membrane is completely impermeable at y_1; i.e.

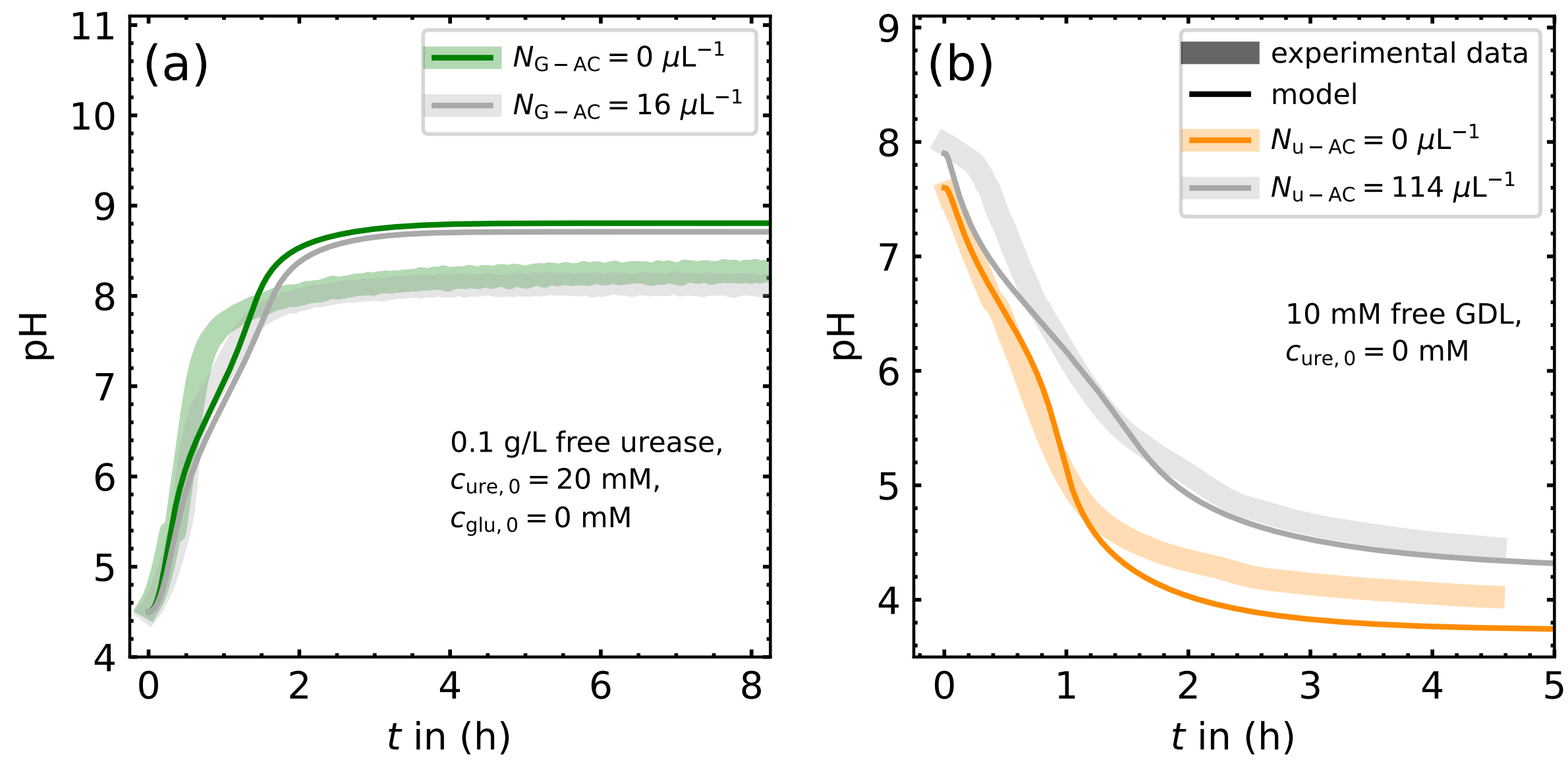

Figure S4: **Buffering effect of the ACs on pH. (a)** pH evolution of free urease, 20 mM urea, and no glucose in a system without ACs (gray) and with GOx-ACs (red). The thick lines correspond to the experimental data [14], the thin lines to the model. **(b)** pH evolution of free GDL and no urea in a system without ACs (gray) and with urease-ACs (red). The experimental data is reproduced from ref [14] used under CC-BY 4.0 license. Copyright 2024 Joshua Krehan et al. (http://creativecommons.org/licenses/by/4.0/).

$P(\mathrm{pH} \leq y_1) = 0$. The data point at pH 7.3 is excluded because it involves the start of the experiment and deviates significantly from the others. We fix $y_1 = 4.6$ because this is the lower pH limit observed in the experiments (see Figure 2a). The other parameters of eq (S4) are fitted and we obtain $A = 3.7 \pm 0.4$, $d_1 = 3.08 \pm 0.22$, $y_2 = 5.73 \pm 0.08$, $d_2 = 0.86 \pm 0.11$. During the same fitting procedure the prefactor of eq (1b) is fitted which leads to $k_{\mathrm{GOx}} = 8.3 \pm 0.7$ mM/h.

The permeability term for the urease-ACs is obtained analogously to the GOx-ACs. Figure S2 shows the pH evolution and the $P(\mathrm{pH})$ data and fit, analogous to Figure 2. As discussed in the context of eq (S1c), the starting time of the urease experiments includes an unknown reducing effect covered via $T(t)$. To avoid introducing a time dependence into the fit, we decided to use only data with pH > 6.4 for the fit. The data for $P(\mathrm{pH})$ shows one sigmoidal decrease. To remove the second sigmoidal term in eq (S4), we fix $A = 0$, which makes y_2 and d_2 irrelevant. In addition, we fix $y_1 = 7.3$, the highest observed pH. This leaves d_1 as the single fit parameter for which we obtain $d_1 = -2.22 \pm 0.23$. The value is negative due to the decrease in permeability with increasing pH. During the same fitting procedure, the prefactor of eq (S1c) is fitted, yielding $k_{\mathrm{urease}} = 6.9 \pm 0.7$ mM/h. The fitted curve describes well the decrease between pH 6.6 and 7.3. This region is crucial because it covers the closing mechanism and the system spends most of the time in this pH range.

S3.3.1 Relation between Permeability Term and AC Diameter

The obtained terms $P(\mathrm{pH})$ in Figure 2b and Figure S2b provide information about the permeability of the two AC-membranes. The permeability terms are compared to the experimentally measured AC diameter $\sigma(\mathrm{pH})$ in Figure S5.

For the GOx-ACs we identify that the second increase in $P(\mathrm{pH})$ corresponds to the swelling of the ACs. In contrast, the first increase occurs without a change in diameter. Thus, we hypothesize structural changes within the polymer membrane. The dependence $P(\sigma)$ in Figure S5e shows a super-exponential behavior for the swelling regime. This is in accordance with hydrogel permeability theory [17]; for a more detailed discussion see ref[18].

For the urease-ACs the decrease in permeability coincides with the AC collapse around pH 6.8. The direct dependency of $P(\sigma)$ in Figure S5f reveals a logarithmic-like increase. This means the membrane is permeable during most of the collapse process and quickly becomes impermeable when approaching the minimum size.

S4 Model Details for Free Enzymes

S4.1 Free Urease in the GOx-AC System

To model the free urease in the protection mechanism in Figure 4a, we use a variation of the model proposed by Ridgway-Brown *et al.* [2]. As in the reaction in the ACs, the urease catalyzes the conversion of urea into ammonia and CO_2. The pH-dependent expression by Ridgway-Brown *et al.* is of the form

$$\dot{c}_{\mathrm{amm}} = -2\dot{c}_{\mathrm{ure}}, \tag{S5a}$$

$$\dot{c}_{\mathrm{CO2}} = -\dot{c}_{\mathrm{ure}}, \tag{S5b}$$

$$\dot{c}_{\mathrm{ure}} = -k_{\mathrm{urease}}^{\mathrm{free}} T(t) \frac{c_{\mathrm{ure}}}{K_{\mathrm{M,urease}} + c_{\mathrm{ure}}} \frac{1}{1 + \frac{[\mathrm{H}^+]}{K_{\mathrm{es1}}} + \frac{K_{\mathrm{es2}}}{[\mathrm{H}^+]}}. \tag{S5c}$$

We added the time-delay prefactor $T(t)$ from eq (S2) in eq (S5c) to account for the initial delay in the pH increase observed in the experiment. The $T(t)$ term does not appear in ref [2]. The prefactor $k_{\mathrm{urease}}^{\mathrm{free}} = (0.0141 \pm 0.0012)/\mathrm{h}$ accounts for system-specific effects (such as the free urease concentration) and is obtained by fitting the model to the experimental data [14] shown in Figure 4a (green line). The model uses the Michaelis-Menten rate with the Michaelis-Menten constant $K_{\mathrm{M,urease}} = 3$ mM [2]. In addition, the pH-dependent activity of urease is multiplied to the rate. This term is identical to the activity term in the main model (see eq (3)).

The model is incorporated into the GOx-AC model by adding two more variables to the system, c_{ure} and c_{amm}. The CO_2 concentration is not a free variable since it fulfills the condition $c_{\mathrm{CO2}} = c_{\mathrm{amm}}/2$. The initial conditions for the concentrations are $c_{\mathrm{ure},0} = 20$ mM as stated in the experimental work [14] and $c_{\mathrm{amm},0} = 0$.

S4.2 Free GDL in the Urease-AC System

To model the free GDL in the protection mechanism in Figure 4b we use the model proposed by Skou *et al.* [6]. The GDL spontaneously hydrolyzes into gluconic acid. The pH-dependent expression by Skou *et al.* is of the form

$$\dot{c}_{\mathrm{GDL}} = -\dot{c}_{\mathrm{ga}}, \tag{S6a}$$

$$\dot{c}_{\mathrm{ga}} = k_{\mathrm{GDL}}^{\mathrm{free}} c_{\mathrm{GDL}} \left(k_{\mathrm{H_2O}} + k_{\mathrm{OH^-}} 10^{\mathrm{pH}-14}\right) \left[1 + \frac{1}{K_\delta (1 + K_{\mathrm{a,GDL}}/10^{-\mathrm{pH}})}\right]. \tag{S6b}$$

The dimensionless prefactor $k_{\mathrm{GDL}}^{\mathrm{free}} = 2.42 \pm 0.04$ accounts for system-specific effects such as local diffusion limitations, and is obtained by fitting the model to the experimental data [14] shown in Figure S4b (orange line). The rate is approximately first-order in c_{GDL} for low concentrations. The other terms describe the pH-dependence with the constants $K_\delta = 3.68$, $K_{\mathrm{a,GDL}} = 2.40 \cdot 10^{-4}$ mol^{-1}L, $k_{\mathrm{H_2O}} = 0.165$ h^{-1}, and $k_{\mathrm{OH^-}} = 9.94 \cdot 10^6$ h^{-1}Lmol^{-1}. All four constants are taken from [6] where the first two constants are literature values and the last two were obtained by fitting their experimental data.

The model is incorporated into the urease-AC model by adding two more variables to the system, c_{GDL} and c_{ga}. The initial conditions for these variables are $c_{\mathrm{GDL},0} = 10$ mM as stated in the experimental work [14] and $c_{\mathrm{ga},0} = 0$.

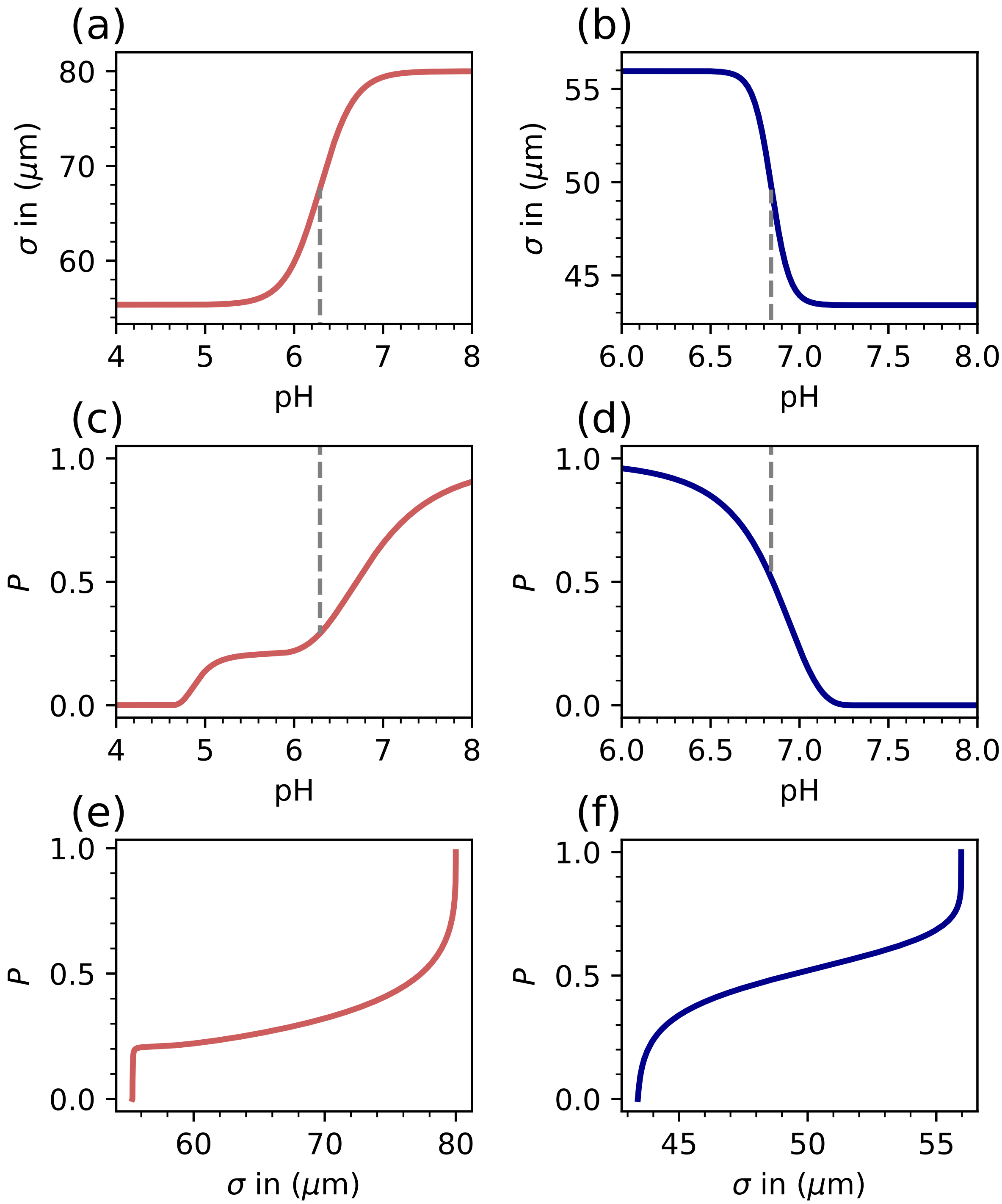

Figure S5: **Relation between AC diameter σ, permeability term P, and pH for both AC types.** **(a)** AC diameter vs pH for GOx-ACs and **(b)** urease-ACs. The dashed lines indicate the centers of the sigmoidal functions. Both data sets are taken from the experiments in ref [14]. **(c)** Permeability term vs pH for GOx-ACs and **(d)** urease-ACs from eq (S4). **(e)** Permeability term vs diameter for GOx-ACs and **(f)** urease-ACs. The experimental data in (a) and (b) are reproduced from ref [14] used under CC-BY 4.0 license. Copyright 2024 Joshua Krehan et al. (http://creativecommons.org/licenses/by/4.0/).

S5 Additional Analysis

S5.1 Required Time for the pH Decrease for Different GOx-AC Concentrations

Intuitively, one might expect that the pH decrease speeds up with the number of GOx-ACs, because more ACs produce more gluconic acid. However, the experiments show the opposite, see Figure 3a and Figure S6a. The reason lies in the interplay between the saturated reaction speed by $\lambda_{\text{G-AC}} = 2.97\ \mu\text{L}^{-1}$ and the AC buffer capacity of 0.11 mM per AC/μL (see Section S3.2). While more ACs do not speed up the gluconic acid production for $N_{\text{G-AC}} \gg \lambda_{\text{G-AC}}$, the AC buffer capacity always increases linearly with $N_{\text{G-AC}}$. For very low $N_{\text{G-AC}}$ the solution buffer capacity of 10 mM dominates and adding more ACs accelerates the pH decrease. Figure S6a shows the modeled pH evolution for different AC concentrations revealing the existence of a minimum in the time needed for the pH decrease. To quantify this, Figure S6b shows the time required to reach pH 5 as a function of $N_{\text{G-AC}}$. We find a minimum required time of 3.9 h for $N^{\text{crit}}_{\text{G-AC}} = 13\ \mu\text{L}^{-1}$. This also indicates that the experimentally chosen concentration of $N_{\text{G-AC}} = 16\ \mu\text{L}^{-1}$ is close to the optimum for a fast pH decrease.

Note that the minimum position shifts to higher AC concentrations for higher amounts of buffer solution. In addition, the minimum position depends also slightly on the chosen critical pH, in this case 5.0, because the acid dissociation constants of the buffer solution and the AC membrane differ.

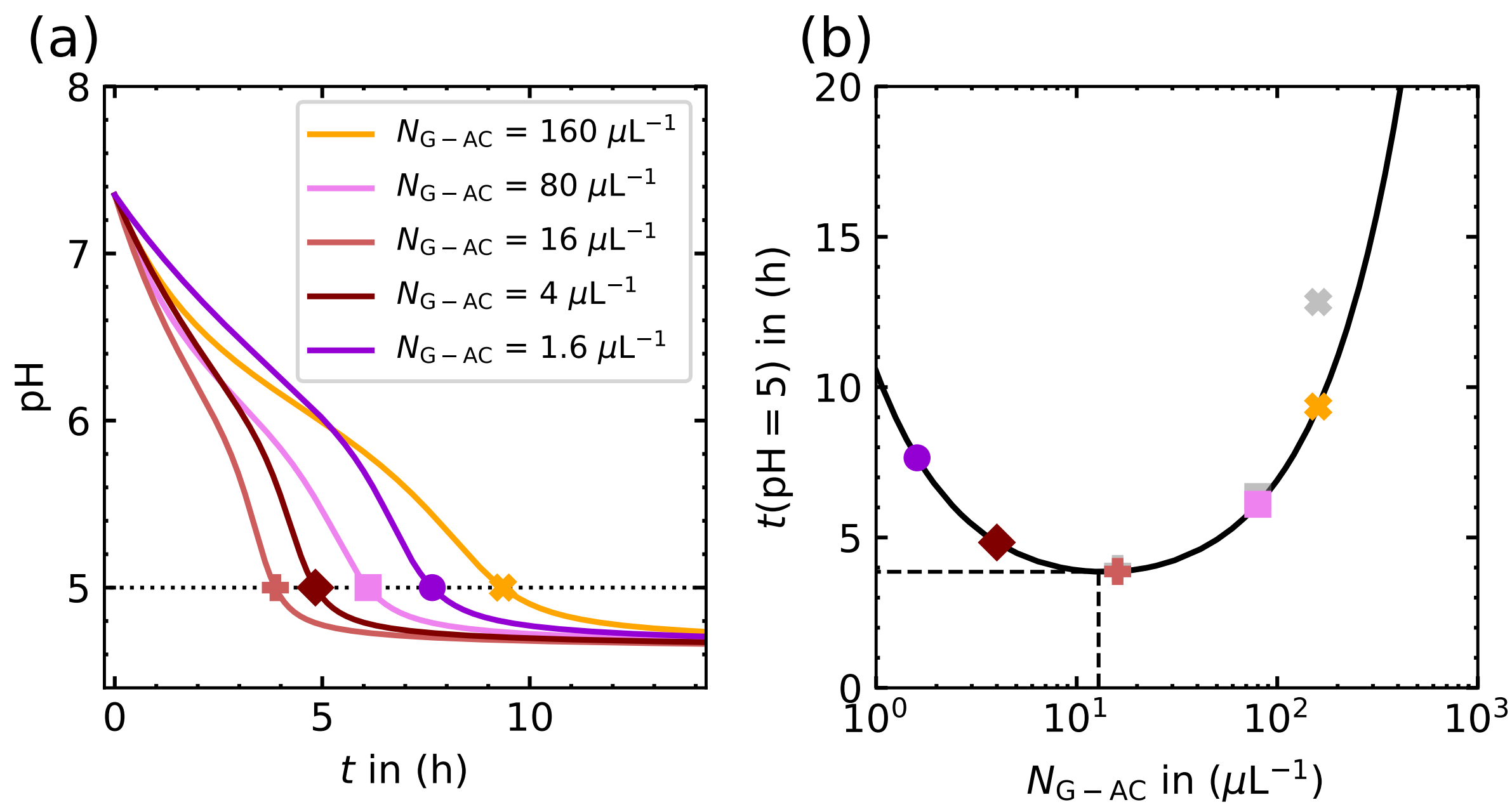

Figure S6: **Time for pH decrease for different GOx-AC concentrations.** **(a)** pH trajectories for different GOx-AC concentrations $c^{\text{GOx}}_{\text{ACs}}$ as in Figure 3a but with two more model lines (1.6, 4 ACs/μL); with $\rho^{\text{in}}_{\text{GOx}} = 10$ g/L, $c_{\text{GLU},0} = 100$ mM. The symbols indicate at which time the trajectories reach pH 5.0 (dotted line). **(b)** Time when the trajectories reach pH 5.0 as a function of the GOx-AC concentration in a log-lin plot. The colored symbols correspond to the symbols in (a). The three gray symbols (plus, square, cross) show the experimental values; the plus and square are obscured by the colored symbols. The dashed lines indicate the minimum at 13 ACs/μL of 3.9 h.

S5.2 Repeated Acid/Base Addition

The experiments show the repeatability of the AC collapse by repeatedly adding a base or acid to the system; see Figure S7. For the GOx-ACs an initial pH of 7.3 was used and the process was started by adding 200 mM glucose. After five hours the pH decreased to 5.0 and the system would converge slowly to pH 4.6. Instead, a strong base, NaOH, is added to the solution which immediately raises the pH back to 7.3. As a consequence the pH decrease starts again. By adding NaOH again after ten and 16 hours it is shown that the cycle can be repeated at least three times.

We model the repeated base addition by slightly changing the pH calculation in eq (2). Since NaOH is a strong base, we only need to modify p_0. The exact amount of base added at each step is unknown. Therefore, we calculate a new p_0 such that we match the new peak in pH from the experiments (5.0h: 7.25, 10.3h: 6.70, 15.6h: 7.18).

Comparing our model with the experimental results in Figure S7a, we observe that our model coincides in general with the measured behavior for the first three cycles. While the final pH values of the cycles match, the model predicts an initially faster pH decrease followed by a slower decrease after four hours. The differences are not large and could be explained by statistical fluctuations of the measurements. It is also possible that there are tiny differences in the AC-behavior after the first cycle. In the fourth cycle the pH decreases in the experiments only to 6.4, which is not covered by our model. One possible explanation is the potentially limited repeatability of the AC-cycle. Another possible explanation is glucose depletion. In our model, 157 mM of the initial 200 mM glucose remains after the fourth cycle, which is enough for many more cycles. In the experiments, measurements reported a consumption of 60 mM during a single cycle. This would result in a glucose shortage in the fourth cycle. At the same time this explanation comes with other problems as discussed in the main article.

This cycle was also experimentally performed with urease-ACs, see Figure S7b. Here, the system started at pH 4.6 and a strong acid (HCl) was added after roughly one, two, and three hours. For the model we modify again p_0 at the according times of the experiment and match the pH with the pH minima from the experiment (1.2h: 4.86, 2.2h: 4.72, 3.2h: 4.68). Since the experiments show the initial slow pH increase in each cycle, we use the time delay function $T(t)$ at the beginning of each cycle. This strengthens the hypothesis that the delay could come from a slow collapse of the urease-AC membrane. In the model-experiment comparison of the repeated cycle of the urease-ACs we observe a very good agreement.

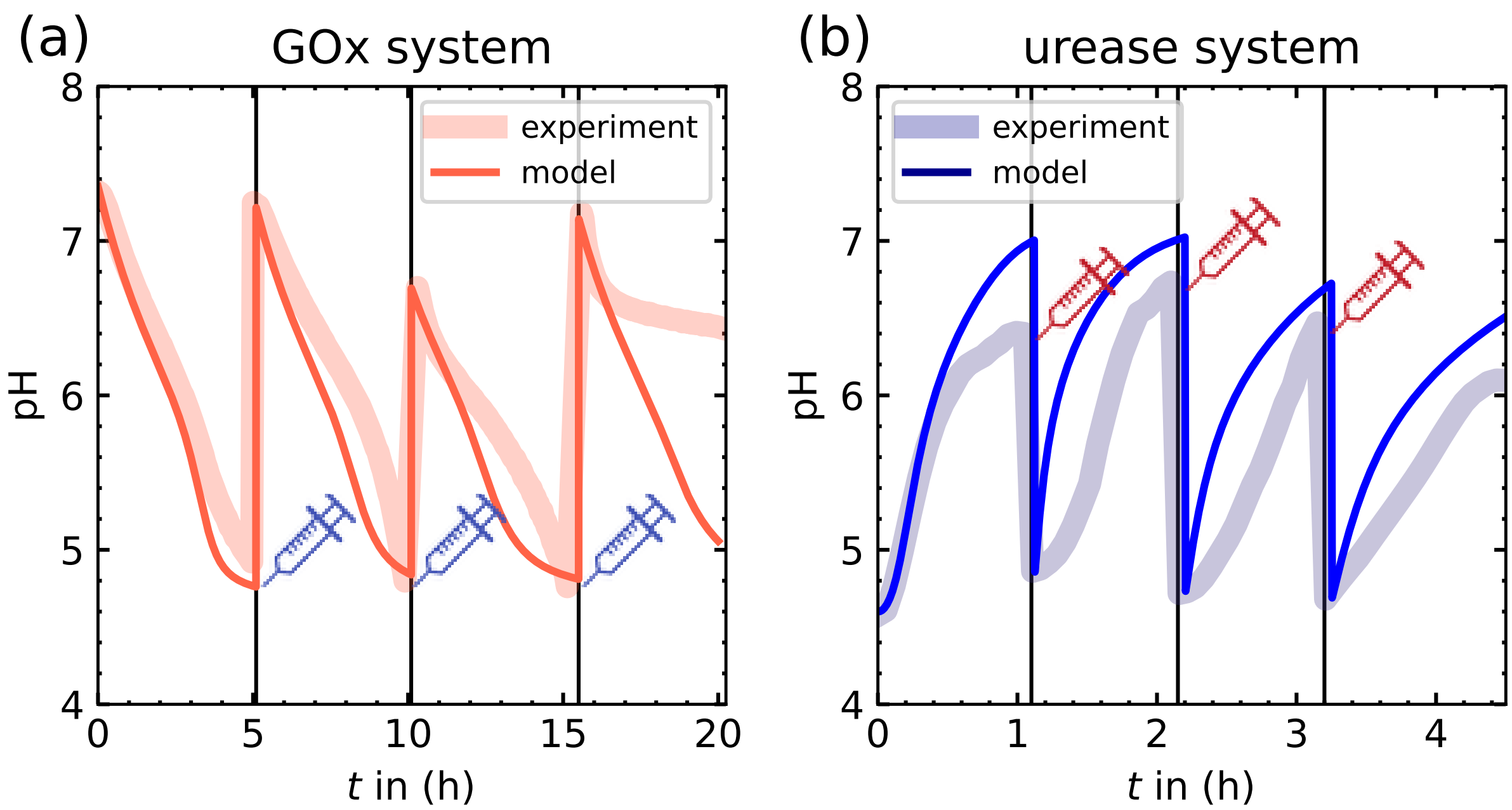

Figure S7: **Repeated acid/base addition. (a)** GOx system where NaOH is added as a base after 5, 10, and 16 hours (indicated by the syringes and vertical black lines). The system runs with $N_{\text{G-AC}} = 16\ \mu\text{L}^{-1}$, $\rho_{\text{GOx}} = 10$ g/L, $c_{\text{glu},0} = 200$ mM. The thick curve is from the experiments [14], the thin curve from our model. **(b)** Urease system where HCl is added as an acid after roughly 1, 2, and 3 hours. The system runs with $N_{\text{u-AC}} = 114\ \mu\text{L}^{-1}$, $\rho_{\text{urease}} = 1$ g/L, $c_{\text{ure},0} = 200$ mM. The experimental data is reproduced from ref [14] used under CC-BY 4.0 license. Copyright 2024 Joshua Krehan et al. (http://creativecommons.org/licenses/by/4.0/).

S6 Analytical Solutions for Fixed Points in AC-Mixtures

To find the analytical solution of the fixed points, we use the definition of φ in eq (4). By setting $\varphi = 0$ we find the solutions where the proton concentration and pH do not change, yielding

$$0 = \frac{\dot{c}_{\mathrm{amm}}(\mathrm{pH}) K_{\mathrm{b,amm}}}{K_{\mathrm{b,amm}} + K_{\mathrm{w}}/[\mathrm{H}^+]} - \frac{\dot{c}_{\mathrm{ga}}(\mathrm{pH}) K_{\mathrm{a,ga}}}{K_{\mathrm{a,ga}} + [\mathrm{H}^+]} - \frac{\dot{c}_{\mathrm{CO2}}(\mathrm{pH}) K_{\mathrm{a,CO2}}}{K_{\mathrm{a,CO2}} + [\mathrm{H}^+]}. \tag{S7}$$

The three time derivatives are the reaction rates of the model, eqs (1b), (S1a), (S1b) for a given $\mathrm{pH} = -\log_{10}([\mathrm{H}^+]/\mathrm{M})$. All solutions of eq (S7) are fixed points.

S6.1 Analytical Solution Solved for the Urease-AC Concentration

Figure 5 shows the fixed points for different urease-AC concentrations $N_{\text{u-AC}}$. To obtain the analytical expression for the fixed points, we solve eq (S7) for $N_{\text{u-AC}}$ which yields

$$N_{\text{u-AC,fixedPoints}} = \lambda_{\text{u-AC}} \frac{r_{\mathrm{G}} \frac{K_{\mathrm{a,ga}}}{K_{\mathrm{a,ga}}+[\mathrm{H}^+]} \frac{N_{\text{G-AC}}}{\lambda_{\text{G-AC}}+N_{\text{G-AC}}}}{r_{\mathrm{u}} \left(2\frac{K_{\mathrm{b,amm}}}{K_{\mathrm{b,amm}}+K_{\mathrm{w}}/[\mathrm{H}^+]} - \frac{K_{\mathrm{a,CO2}}}{K_{\mathrm{a,CO2}}+[\mathrm{H}^+]} \right) - r_{\mathrm{G}} \frac{K_{\mathrm{a,ga}}}{K_{\mathrm{a,ga}}+[\mathrm{H}^+]} \frac{N_{\text{G-AC}}}{\lambda_{\text{G-AC}}+N_{\text{G-AC}}}} \tag{S8a}$$

with

$$r_{\mathrm{G}} = k_{\mathrm{GOx}} a(\mathrm{pH}) P(\mathrm{pH}) B_{\mathrm{ref}} \frac{\rho_{\mathrm{GOx}}}{\lambda_{\mathrm{GOx}} + \rho_{\mathrm{GOx}}} \frac{c_{\mathrm{glu}}}{\lambda_{\mathrm{glu}} + c_{\mathrm{glu}}}, \tag{S8b}$$

$$r_{\mathrm{u}} = k_{\mathrm{urease}} a(\mathrm{pH}) P(\mathrm{pH}) B_{\mathrm{ref}} \frac{\rho_{\mathrm{urease}}}{\lambda_{\mathrm{urease}} + \rho_{\mathrm{urease}}} \frac{c_{\mathrm{ure}}}{\lambda_{\mathrm{ure}} + c_{\mathrm{ure}}}. \tag{S8c}$$

The green line in Figure 5b is generated by plotting $N_{\text{u-AC,fixedPoints}}(\mathrm{pH})$ using $\mathrm{pH} = -\log_{10}([\mathrm{H}^+]/\mathrm{M})$. The stability condition $\partial N_{\text{u-AC,fixedPoints}}/\partial \mathrm{pH} > 0$ is determined numerically by locating the local minimum and maximum in $N_{\text{u-AC,fixedPoints}}(\mathrm{pH})$.

The state diagram in Figure 5c is obtained by numerically calculating the local minimum and maximum of $N_{\text{u-AC,fixedPoints}}(\mathrm{pH}; N_{\text{G-AC}})$ for 500 values of $N_{\text{G-AC}}$.

S6.2 Analytical Solution Solved for the Urea Concentration

Figure 6 shows the fixed points for different urea concentrations c_{ure}. To obtain the analytical expression for the fixed points, we solve eq (S7) for c_{ure} which yields

$$c_{\mathrm{ure,fixedPoints}} = \lambda_{\mathrm{ure}} \frac{\tilde{r}_{\mathrm{G}} \frac{K_{\mathrm{a,ga}}}{K_{\mathrm{a,ga}}+[\mathrm{H}^+]} \frac{c_{\mathrm{glu}}}{\lambda_{\mathrm{glu}}+c_{\mathrm{glu}}}}{\tilde{r}_{\mathrm{u}} \left(2\frac{K_{\mathrm{b,amm}}}{K_{\mathrm{b,amm}}+K_{\mathrm{w}}/[\mathrm{H}^+]} - \frac{K_{\mathrm{a,CO2}}}{K_{\mathrm{a,CO2}}+[\mathrm{H}^+]} \right) - \tilde{r}_{\mathrm{G}} \frac{K_{\mathrm{a,ga}}}{K_{\mathrm{a,ga}}+[\mathrm{H}^+]} \frac{c_{\mathrm{glu}}}{\lambda_{\mathrm{glu}}+c_{\mathrm{glu}}}} \tag{S9a}$$

with

$$\tilde{r}_{\mathrm{G}} = k_{\mathrm{GOx}} a(\mathrm{pH}) P(\mathrm{pH}) B_{\mathrm{ref}} \frac{N_{\text{G-AC}}}{\lambda_{\text{G-AC}} + N_{\text{G-AC}}} \frac{\rho_{\mathrm{GOx}}}{\lambda_{\mathrm{GOx}} + \rho_{\mathrm{GOx}}}, \tag{S9b}$$

$$\tilde{r}_{\mathrm{u}} = k_{\mathrm{urease}} a(\mathrm{pH}) P(\mathrm{pH}) B_{\mathrm{ref}} \frac{N_{\text{u-AC}}}{\lambda_{\text{u-AC}} + N_{\text{u-AC}}} \frac{\rho_{\mathrm{urease}}}{\lambda_{\mathrm{urease}} + \rho_{\mathrm{urease}}}. \tag{S9c}$$

Analogous to Figure 5b, the green line in Figure 6b is generated by plotting $c_{\mathrm{ure,fixedPoints}}(\mathrm{pH})$ using $\mathrm{pH} = -\log_{10}([\mathrm{H}^+]/\mathrm{M})$. The stability condition $\partial c_{\mathrm{ure,fixedPoints}}/\partial \mathrm{pH} > 0$ is calculated numerically by finding the local minimum and maximum in $c_{\mathrm{ure,fixedPoints}}(\mathrm{pH})$.

The state diagram in Figure 6c is obtained by numerically calculating the local minimum and maximum of $c_{\text{ure,fixedPoints}}(\text{pH}; c_{\text{glu}})$ for 1000 values of c_{glu}.

References

(1) Bright, H. J.; Appleby, M. The pH Dependence of the Individual Steps in the Glucose Oxidase Reaction. *J. Biol. Chem.* **1969**, *244*, 3625–3634.

(2) Ridgway-Brown, D.; Leathard, A. S.; France, O.; Muench, S. P.; Webb, M. E.; Jeuken, L. J. C.; Henderson, P. J. F.; Taylor, A. F.; Beales, P. A. Membrane Transport Modulates the pH-Regulated Feedback of an Enzyme Reaction Confined within Lipid Vesicles. *ACS Nano* **2025**, *19*, 9814–9825.

(3) Schüwer, N.; Klok, H.-A. Tuning the pH Sensitivity of Poly(methacrylic acid) Brushes. *Langmuir* **2011**, *27*, 4789–4796.

(4) Schmalz, A.; Hanisch, M.; Schmalz, H.; Müller, A. H. Double stimuli-responsive behavior of linear and star-shaped poly(N,N-diethylaminoethyl methacrylate) in aqueous solution. *Polymer* **2010**, *51*, 1213–1217.

(5) Chen, Q.; Li, S.; Feng, Z.; Wang, M.; Cai, C.; Wang, J.; Zhang, L. Poly(2-(diethylamino)ethyl methacrylate)-based, pH-responsive, copolymeric mixed micelles for targeting anticancer drug control release. *Int. J. Nanomed.* **2017**, *Volume 12*, 6857–6870.

(6) Skou, E. M.; Jacobsen, T.; Convert, P.; Beyer, L.; Bastiansen, O.; Braathen, G.; Fernholt, L.; Gundersen, G.; Nielsen, C. J.; Cyvin, B. N.; Cyvin, S. J. The Hydrolysis Kinetics of Glucono-delta-lactone in Solutions without Anion Catalysis and a Systematical Error in Weakly Buffered pH-static Kinetics. *Acta Chem. Scand.* **1982**, *36a*, 417–422.

(7) Heuser, T.; Weyandt, E.; Walther, A. Biocatalytic Feedback-Driven Temporal Programming of Self-Regulating Peptide Hydrogels. *Angew. Chem., Int. Ed.* **2015**, *54*, 13258–13262.

(8) Krajewska, B.; Ciurli, S. Jack bean (Canavalia ensiformis) urease. Probing acid–base groups of the active site by pH variation. *Plant Physiol. Biochem.* **2005**, *43*, 651–658.

(9) Krajewska, B. A combined temperature-pH study of urease kinetics. Assigning pKa values to ionizable groups of the active site involved in the catalytic reaction. *J. Mol. Catal. B Enzym.* **2016**, *124*, 70–76.

(10) Hu, G.; Pojman, J. A.; Scott, S. K.; Wrobel, M. M.; Taylor, A. F. Base-Catalyzed Feedback in the Urea - Urease Reaction. *J. Phys. Chem. B* **2010**, *114*, 14059–14063.

(11) Markovic, V. M.; Bánsági, T.; McKenzie, D.; Mai, A.; Pojman, J. A.; Taylor, A. F. Influence of reaction-induced convection on quorum sensing in enzyme-loaded agarose beads. *Chaos* **2019**, *29*, 033130.

(12) Singh, M.; Ungku Faiz, U. Z. A.; Gravelsins, S.; Suganuma, Y.; Kotoulas, N. K.; Croxall, M.; Khan-Trottier, A.; Goh, C.; Dhirani, A.-A. Glucose oxidase kinetics using MnO 2 nanosheets: confirming Michaelis–Menten kinetics and quantifying decreasing enzyme performance with increasing buffer concentration. *Nanoscale Adv.* **2021**, *3*, 3816–3823.

(13) Krajewska, B.; Zaborska, W. The effect of phosphate buffer in the range of pH 5.80–8.07 on jack bean urease activity. *J. Mol. Catal. B Enzym.* **1999**, *6*, 75–81.

(14) Krehan, J.; Li, C.-R.; Masukawa, M.; Amstad, E.; Walther, A. Homeostatic artificial cells enable self-protection in prototissue spheroids. *Chem* **2025**, *11*, 102409.

(15) Dulbecco, R.; Vogt, M. PLAQUE FORMATION AND ISOLATION OF PURE LINES WITH POLIOMYELITIS VIRUSES. *J. Exp. Med.* **1954**, *99*, 167–182.

(16) Werner, J. G.; Nawar, S.; Solovev, A. A.; Weitz, D. A. Hydrogel Microcapsules with Dynamic pH-Responsive Properties from Methacrylic Anhydride. *Macromolecules* **2018**, *51*, 5798–5805.

(17) Gehrke, S. H.; Fisher, J. P.; Palasis, M.; Lund, M. E. Factors Determining Hydrogel Permeability. *Ann. N.Y. Acad. Sci.* **1997**, *831*, 179–184.

(18) Milster, S.; Darwish, A.; Göth, N.; Dzubiella, J. Synergistic chemomechanical dynamics of feedback-controlled microreactors. *Phys. Rev. E* **2023**, *108*, L042601.
